# Leveraging the Role of the Metastatic Associated Protein Anterior Gradient Homologue 2 in Unfolded Protein Degradation: A Novel Therapeutic Biomarker for Cancer

**DOI:** 10.3390/cancers11070890

**Published:** 2019-06-26

**Authors:** Reem Alsereihi, Hans-Juergen Schulten, Sherin Bakhashab, Kulvinder Saini, Ahmed M. Al-Hejin, Deema Hussein

**Affiliations:** 1 Neurooncology Translational Group, King Fahd Medical Research Center, King Abdulaziz University, P.O. Box 80216, Jeddah 21589, Saudi Arabia; 2 Department of Biological Sciences, Faculty of Science, King Abdulaziz University, P.O. Box 80203, Jeddah 21589, Saudi Arabia; 3 Center of Excellence in Genomic Medicine Research, King Abdulaziz University, P.O. Box 80216, Jeddah 21589, Saudi Arabia; 4 Department of Medical Laboratory Technology, Faculty of Applied Medical Sciences, King Abdulaziz University, Jeddah 21589, Saudi Arabia; 5 Biochemistry Department, King Abdulaziz University, P.O. Box 80218, Jeddah 21589, Saudi Arabia; 6 School of Biotechnology, Eternal University, Baru Sahib-173101, Himachal Pradesh, India; 7 Microbiology Unit, King Fahad Medical Research Center, King Abdulaziz University, P.O. Box 80216, Jeddah 21589, Saudi Arabia

**Keywords:** anterior gradient homologue 2, genomic integrity, proliferation, apoptosis, angiogenesis, metastasis, adhesion, migration, stemness, inflammation, unfolded protein response

## Abstract

Effective diagnostic, prognostic and therapeutic biomarkers can help in tracking disease progress, predict patients’ survival, and considerably affect the drive for successful clinical management. The present review aims to determine how the metastatic-linked protein anterior gradient homologue 2 (AGR2) operates to affect cancer progression, and to identify associated potential diagnostic, prognostic and therapeutic biomarkers, particularly in central nervous system (CNS) tumors. Studies that show a high expression level of AGR2, and associate the protein expression with the resilience to chemotherapeutic treatments or with poor cancer survival, are reported. The primary protein structures of the seven variants of AGR2, including their functional domains, are summarized. Based on experiments in various biological models, this review shows an orchestra of multiple molecules that regulate AGR2 expression, including a feedback loop with p53. The AGR2-associated molecular functions and pathways including genomic integrity, proliferation, apoptosis, angiogenesis, adhesion, migration, stemness, and inflammation, are detailed. In addition, the mechanisms that can enable the rampant oncogenic effects of AGR2 are clarified. The different strategies used to therapeutically target AGR2-positive cancer cells are evaluated in light of the current evidence. Moreover, novel associated pathways and clinically relevant deregulated genes in AGR2 high CNS tumors are identified using a meta-analysis approach.

## 1. Introduction

The anterior gradient homolog 2 protein (AGR2) is considered a member of the protein disulphide isomerase (PDI) superfamily. This gene is also known as AG2, HAG2 and PDIA17 and is a homologue of the secreted cement gland gene XAG-2 [1]. Several studies have been conducted to unravel the role of AGR2 in different biological models. The expression of the AGR2 homolog XAG-2 was initially observed in *Xenopus laevis*, particularly in the dorso-anterior ectoderm region forming the cement gland, which is important for the proper formation of the forebrain [2]. XAG-2 has been shown to affect the adhesive-like property of the cement gland, a feature that has been associated with the metastasis of mammalian cancers [3,4,5]. Studies have also identified the role of the homolog of AGR2 (NAG) in limb regeneration and in the development of amphibians [6,7]. The outcomes from this work, together with experiments in mammalian cells, showed an inverse correlation between AGR2 and p21 expression [8,9], highlighting a possible growth-modulating function of AGR2. More importantly, null AGR2 transgenic mice were shown to have defects in mucin production and a susceptibility to toxin-stimulated inflammatory bowel disease (IBD) [10]. In parallel, several cellular studies showed that AGR2 resided mainly in the endoplasmic reticulum (ER), and thus AGR2 was suggested to act as a chaperone, operating especially in the conditions of ER stress [1,10]. Interestingly, extracellular AGR2 protein (eAGR2) was shown to accelerate the wound-healing process following skin damage by increasing the migration of keratinocytes and fibroblasts near the wounded areas [11]. Recently, the dimerized AGR2 was demonstrated to act as a sensor of ER homeostasis and was shown to correlate with the severity of inflammatory bowel disease [12]. In addition, secreted AGR2 was shown to be able to selectively promote monocyte attraction.

The clinical relevance of AGR2 overexpression in cancer progression was particularly emphasized when the human AGR2 protein was shown to attenuate the p53 activity following DNA damage through the suppression of phosphorylated p53 [8]. The AGR2 gene was identified later in a gene expression profiling study in the MDA-MB-231 breast cancer cell line to promote survival and invasion under stressed and hypoxic conditions [13]. The suppression of AGR2 was subsequently shown in several cell lines to increase the expression of wild-type p53 possibly though the upregulation of DUSP10 [14,15,16,17]. The expression and activity of a number of proteins have since been shown to be modulated by the presence of AGR2 [18,19]. Several functional domains have been identified that contribute to its function, particularly its Cys81 consensus motif, which is important for the peptide-binding activity of the AGR2 protein [20,21].

Gene expression signatures have shown that AGR2 is overexpressed in many tumors. Recently, the AGR2 expression was systematically analyzed in solid tumors using STATA 12.0. A total of 20 studies containing 3,285 cases were included. Tumor data for prostate cancer, breast cancer, lung cancer, colorectal cancer, ovarian cancer and gastric cancer were analyzed. The results showed that the AGR2 overexpression had a negative effect on the overall survival (OS) and time to tumor progression (TTP) of patients with a solid tumor. In particular, the analysis for breast cancer patients found the AGR2 overexpression to be significantly associated with poor OS and TTP [22]. However, this meta-analysis did not include central nervous system (CNS) tumors, even though the AGR2 overexpression had been detected in CNS tumors, including glioblastoma multiforme (GBM) and meningiomas [23,24,25]. 

The association of AGR2 with multiple cancer-related pathways, particularly the metastatic pathway, combined with its nature to be secreted into the extracellular space makes it a tantalizing non-invasive cancer biomarker. In this review, the recent findings associated with AGR2, its structure, regulations, associated molecular functions and mechanisms that are predicted to enable the oncogenic function of AGR2 are presented. Using a meta-analysis approach, the identified signal transduction pathways modulated in the CNS tumors overexpressing AGR2 are shown. 

## 2. Molecular Structure

Three structurally related AGR proteins have been discovered, namely, AGR1, AGR2 and AGR3. Initially, all three were grouped as a family because of their high similarity to the classic thioredoxin fold containing the CXXC motif. AGR3, the third member of the AGR family, was initially identified from multiple human breast tumors [26] and was found to be overexpressed in estrogen receptor-negative human ovarian cancers [27]. However, further studies accentuated the function of AGR2 and deemphasized the role of other family members, particularly in relation to cancer. In addition, several studies confirmed the clear functional differences among all three proteins [28]. 

**Figure 1:**
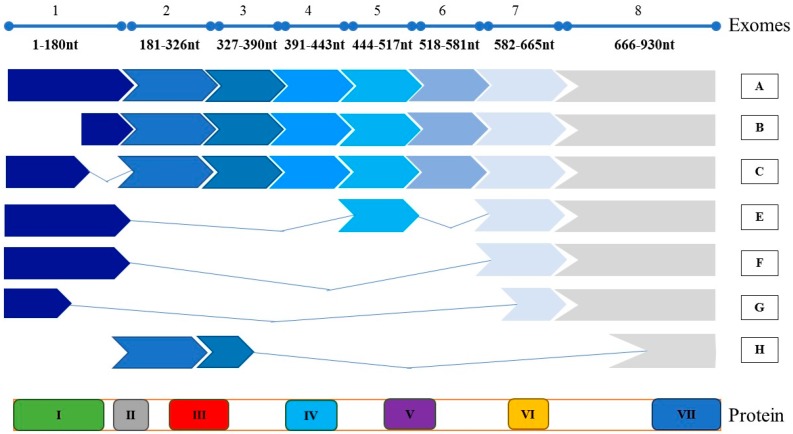
Illustrations of the AGR2 gene and its corresponding protein structure, as well as the detected splice variants. A-H represent AGR2 splice variants. Functional domains for AGR2 protein are shown as: (I) 1-20aa CHPS, directs the AGR2 protein to the ER and contains block of hydrophobic amino acid residues; (II) 20-21aa Ala-Lys, is a leader sequence; (III) 21-45aa, is important for the extracellular secretion; (IV) 60-64aa, is required for dimerization; (V) CXXS motif, interacts with intermediates in redox reactions during folding and retrograde transport in the secretory pathway; (VI) A peptide binding lobe that is important for protein–protein interactions; and (VII) KETL, works as an ER retention signal for targeting proteins into the secretory pathway and to traffic AGR2 protein to different compartments in the cell.

The AGR2 gene was mapped to chromosome 7p21.1 [29]. The intracellular encoded protein has been detected to have a molecular weight of 19 kDa [30,31], with a fully mature AGR2 protein being 175 aa long. The AGR2 protein has an N-terminal ER signal sequence and a C-terminal ER retention sequence [18]. Several isoforms of AGR2 have been identified, Figure 1. The splice variants of AGR2 were found to be expressed in certain tissues or in distinct neoplastic regions [32]. Aside from the wild-type AGR2 transcripts A and B, other variants, namely, C, E, F, G and H, were also detected in the prostate cancer cell line PC-3 [5,32]. All variants of AGR2 contain the KETL region, which functions as an ER retention signal and is vital for targeting the protein into the secretory pathways, and for trafficking into different compartments of the cell [33]. Apart from variant H, all detected variants have a peptide binding loop important for protein–protein interactions and a CHPS domain, which is required to direct the AGR2 protein to the ER [34]. Moreover, variants A, B and C have domains II, III, IV and V; variant E has domain V; and variant H has domains II and III. Domain II is an Ala-Lys leader sequence, domain III is important for the extracellular secretion [12,32], domain IV is required for dimerization [20,35] and domain V contains a CXXS motif that interacts with intermediates in redox reactions during folding and retrograde transport in the secretory pathway. More importantly, a variant from of the AGR2 protein was shown to have the ability to be secreted and O-glycosylated by the breast cancer MCF7 cell line [36]. 

Recently, work carried out using HEK293T cells, transfected with the ER Mammalian protein–protein Interaction Trap bait/prey system, identified 42 enhancer candidates and 29 inhibitor candidates of AGR2 homodimerisation. In this work, constitutively monomeric AGR2 with deletion at E60A was secreted more efficiently than wild type AGR2, while AGR2 molecules deleted at D45 were retained inside the cell. Interestingly, the secretion of wild type AGR2 depended on the interaction of AGR2 at sites K66 and Y111 with the transmembrane p24 trafficking protein 2 (TMED2) [12]. Thus, it is likely that the structures for isoforms that are able to be secreted into the extracellular space resemble that of isoform H. This is consistent with the observation that AGR2 SV-H was the most significantly expressed isoform in urine exosomes isolated from prostate cancer patients [32]. However, the exact mechanism for AGR2 secretory transportation remains to be clarified [12].

## 3. Expression and Survival 

The AGR2 expression has been detected in both healthy and cancer cells. In healthy human tissues, the RNA-seq expression patterns of AGR2 were identified only in specific tissues [37]. In particular, AGR2 was shown to be expressed in the stomach, colon, duodenum, small intestine, gall bladder, prostate, urinary bladder and lung tissues [37]. Several independent studies assessed this expression in different tissues. The assessment of the AGR2 expression levels using immunohistochemistry (IHC) indicated a high expression in 28 normal intestinal samples and in normal colon cells [38]. Both the AGR2 mRNA expression and the protein levels were shown to be highly expressed in normal thyroids samples [39]. Interestingly, the AGR2 protein expression was shown to be particularly and predominantly expressed in glandular cells found in the aforementioned organs [40,41,42]. Perhaps such expression patterns suggest a natural alerting role of AGR2 in response to the continuous exposure to damage by microbes, ingested toxins or chemicals that include drugs and food additives. Outcomes in AGR2 studies for inflammatory bowel disease support this notion [12].

The high expression of AGR2 has also been detected in many cancers, including those arising in the breast, respiratory, reproductive, urinary, digestive and endocrine systems. In stages I and II tumors of different breast cancer types, 232 out of 351 breast cancers (66%) treated with adjuvant hormonal therapy showed positive staining of AGR2 [43]. The low expression of the protein was detected in the estrogen receptor-negative cases, whereas the high expression was associated with the estrogen receptor-positive cases. More importantly, tumors with a high expression of AGR2 showed a significant trend of a progressive decrease in patients’ survival [43]. In a 20-year follow-up study, an immunocytochemical analysis of 315 stages I and II tumors of breast cancer patients separated samples into positive or negative AGR2 expressed tissues. Out of the 107 patients whose samples showed a negative AGR2 staining, 103 survived (96%) for 20 years in comparison with only 25 out of the 208 patients (26%) whose samples showed a positive AGR2 staining [44]. Correspondingly, the analysis of the data collected from the Oncomine Cancer Microarray Database showed that the AGR2 expression level increased in human epidermal growth factor receptor 2 (HER2)-positive breast tumors [44]. In one study analyzing the protein expression in 69 samples from the Gene Expression Omnibus (GEO) database (48 invasive ductal carcinomas) from non-triple-negative breast cancers (TNBC) patients and 16 TNBC patients and in 622 samples from the Cancer Genome Atlas, AGR2 was found to be highly expressed in non-TNBC that were positive for estrogen receptor, progesterone receptor, or HER2. Correspondingly, survival was enhanced for patients with grade I or II tumors regardless of the tumor type [45].

In pancreatic ductal adenocarcinomas (PDAC), AGR2 mRNA was found to be in mean 14-fold higher expressed in 56 out of 57 pancreatic cancer tissues (98%) than in normal and pancreatitis tissues [46]. In another study, 109 out of 148 samples (74%) from pancreatic cancer patients had highly expressed AGR2 protein, with an elevated expression in female patients [47], and 139 out of 195 PDAC tissue samples (72%) had a high expression of AGR2 [48]. Similarly, gene expression analysis revealed a significant upregulation of AGR2 in the metastatic cholangiocarcinoma KKU-213L5 cell line [49]. In examining the AGR2 expression in PDAC-related tissues using tissue microarrays, six out of eight fibrolamellar carcinomas samples and three out of four metastatic fibrolamellar carcinomas samples had a high expression [50]. In another IHC study, staining showed that AGR2 expression was detected in one case of fibrolamellar carcinoma expressed in 14 cases of mucus-excreting intrahepatic cholangiocarcinomas [51]. 

In prostate cancer, AGR2 expression in a tissue microarray assay was revealed to be increased in 66 prostate cancer adenocarcinoma tissues compared with normal prostatic glandular epithelium tissue [52]. Using antibodies P1G4 (IgG1) and P3A5 (IgG2a) that were generated against the AGR2 protein, a high expression of AGR2 was revealed in prostate tumor specimen tissues and cultured cells [53]. AGR2 was also detected at elevated levels in the urine samples of prostate cancer patients [54].

In ovarian-related cancers, the plasma concentration in 46 samples of patients with ovarian cancer and 61 controls were tested for the immunoreactivity of AGR2, and the results revealed significantly high levels of AGR2 in the cancer cases [55]. The upregulation of AGR2 protein was also seen in low-grade endometrial tumors, which were positively associated with a high expression of hormone receptors [56].

Studies on lung adenocarcinoma samples showed variable levels of AGR2 expression. Using immunostaining, 63 out of 95 non-small cell lung cancer (NSCLC) samples (66%) were demonstrated to have an elevated AGR2 expression [57]. In an IHC study on 60 adenocarcinoma (AdC) samples, AGR2 expression was shown to be between moderate and high in AdC cases [58]. Comparing AGR2 protein levels in 111 primary lung AdC patient samples with 46 non-cancer samples revealed a significantly higher expression in the AdC samples [59]. An increased AGR2 expression was also illustrated in 147 cases of surgically resected lung adenocarcinomas that had epidermal growth factor receptor (EGFR) gene mutations [60]. Interestingly, the expression of AGR2 protein was positively correlated with the expression of the transforming growth factor-alpha in human lung AdC with EGFR mutations [61]. The level of AGR2 expression was particularly meaningful in predicting a better prognosis in younger lung AdC patients [62]. Similarly, AGR2 protein was significantly elevated in both serum and tissue samples of pharyngeal nasal cancers, and the increased levels were associated with tumor node metastasis and relapse along with the low survival rates of patients [63]. Likewise, in Barrett esophagus, the AGR2 expression was elevated in 125 AdC biopsy samples [64].

In gastric cancer samples, AGR2 expression was examined in 436 cases, and the results indicated an elevated AGR2 expression in 204 of the 436 samples (47%). Patients with a low level of AGR2 expression significantly survived longer than those with a high AGR2 expression [65]. In 54 colorectal cancer samples and 19 controls, the results obtained through a real-time polymerase chain reaction (RT-PCR) significantly revealed an enhanced expression of AGR2 mRNAs compared with the controls [66]. Correspondingly, IHC analyses showed that 833 out of the 1,068 tissue samples (78%) of colorectal carcinomas were positive for AGR2 [38]. In a biliary tract cancer study on the SNU-245 and SNU-478 primary patient cell lines, low levels of AGR2 were correlated with increased disease-free survival (DFS), whereas high levels of AGR2 showed a lower DFS [67,68]. In the IHC evaluation of biliary tract tumors, IHC similarly detected AGR2 expression in ductal epithelial cells in 95 out of 100 cancer tissues of the biliary tract [69]. Furthermore, tissue microarray analysis showed a high expression of AGR2 in 36 out of 152 tissues (24%) collected from patients with urothelial bladder carcinoma [69].

AGR2 has also been shown to be overexpressed in other tumors. In the papillary thyroid carcinoma TPC-1 cells, AGR2 was shown to be overexpressed using IHC and quantitative RT-PCR [39]. In the head and neck squamous cell carcinoma (HNSCC) CAL27 cell line, AGR2 protein was highly expressed and shown to be overexpressed in the corresponding cancer stem cells (CSC) [70]. The AGR2 protein was also shown to be highly expressed in pituitary adenoma (PA). The AGR2 expression levels were examined in 117 PA tissue samples using IHC and Western blotting, and 59 patients (51%) were shown to have an overexpressed AGR2 levels [71]. In a different study, AGR2 was variably detected in the serum of 163 PA patients using ELISA [72].

In CNS tumors, the evaluation of AGR2 expression levels in the glioblastomas (GBM) U87 and LN18 cell lines revealed an up-regulated AGR2 [24]. A high expression of AGR2 was also detected in the glioblastoma cell lines T98G, A172, U87 and U251 [25]. In situ and in vitro analyses of meningioma tissue and cell lines showed a high expression of AGR2, particularly in aggressive tumors [23].

## 4. AGR2 Regulation

**Figure 2:**
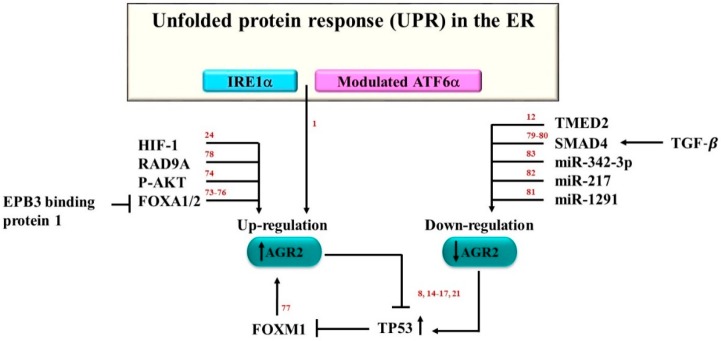
The expression of AGR2 is regulated by multiple proteins. IRE1α, modulated ATF6α, HIF-1α, RAD9A, phosphorylated-AKT, FOXA1/2 have all been shown to contribute to the upregulation of AGR2. In contrast, the upregulation of SMAD4, miR-342-3p, miR-217, miR-1291, and TMED2 have been shown to contribute to the downregulation of AGR2. While p53, and FoxM1 appear to be involved in a feedback regulatory loop. The numbers in red indicate references.

Several molecules that deregulate the expression of AGR2 are shown in Figure 2. In particular, protein expression was shown to be up-regulated following ER stress [1,10,73]. The protein expression level of AGR2 was shown to be affected by both the endonuclease inositol-requiring enzyme1 (IRE1α) and the activating transcription factor 6 (ATF6α) [1], which are pillars of the unfolded protein response (UPR) in the ER machinery. This was demonstrated when IREα and ATF6α were knocked down using small interfering RNA (siRNA) in HeLa cells, resulting in the decreased expression levels of the AGR2 protein [1]. In another study, 3-phosphoinositide-dependent protein kinase1 (PDPK1) and AKT serine/threonine kinase 1 (AKT1) were identified as AGR2 protein triggers, and AKT1 was shown to initiate AGR2 expression [74]. The transcriptional factors forkhead box A1 and 2 (FOXA1 and FOXA2) were shown to upregulate the expression of the AGR2 gene in the LNCaP prostate cancer cell line. By contrast, the ErbB3 binding protein 1 (EBP1) indirectly inhibited AGR2 expression by downregulating the activities of FOXA1 and FOXA2 [75]. Similarly, in the HEK293 cell line, FOXA1 and FOXA2 were shown to upregulate the AGR2 protein level [76]. Forkhead box M1 (FOXM1) was also shown to activate the human AGR2 gene promoter in the pulmonary invasive mucinous adenocarcinoma A539 and H2122 cell lines [77]. In GBM cells, hypoxia-induced factor-1 (HIF-1) was found to play an important role in cell growth through the up-regulation of the AGR2 protein [24]. Another protein that may regulate AGR2 is the RAD9 checkpoint clamp component A (RAD9A). The expression of the AGR2 protein in the prostate cancer cell line PC-3 was shown to be controlled by RAD9A through its specific binding to the 5ʹ-untranslated region of AGR2, and the knockdown of RAD9A resulted in the downregulation of AGR2 [78]. The expression of AGR2 is also affected, in a feedback loop mechanism, by the expression of p53 and its phosphorylation [8,14,15,16,17,21]. 

Several molecules have been associated with the downregulation of AGR2. Transforming growth factor beta (TGF-β) was shown to downregulate AGR2. A study revealed that the induction of the TGF-β SMAD regulatory pathway in the lung adenocarcinoma cell line A549, the breast cancer cell line BT-474, and the pancreatic adenocarcinoma cell line PANC-1, resulted in the significant downregulation of the levels of AGR2 [79]. Pre-treated A549 and PANC-1 cell lines with the inhibitor PD98059, which inhibits both mitogen-activated kinase-like protein and TGF-β, prevented the downregulation of the AGR2 protein level [79]. In PDAC, TGF-β overexpression was also shown to downregulate AGR2 and to decrease the metastatic-associated protein mucin1 (MUC1) through the SMAD family member 4 activation [80]. Several microRNA molecules have been shown to downregulate AGR2. In PANC-1, AGR2 expression was revealed to be significantly downregulated through the actions of microRNA-1291 (miR-1291) [81]. In the chronic myelogenous leukemia (CML) cell line K562, microRNA-217 (miR-217) had a negative effect on AGR2 protein expression. Tyrosine kinase inhibitor treatment was shown to induce drug resistance in the dasatinib-resistant K562 (K562DR) cell line, which decreased the miR-217 level and upregulated the AGR2 protein level [82]. The analysis of the AGR2 protein in 10 NSCLC cell lines revealed that microRNA-342-3p (miR-342-3p) negatively correlated with AGR2 and could directly inhibit AGR2 [83]. Recently, the overexpression of TMED2 was also shown to reduce the expression levels of AGR2 [12]. In sum, the observations indicate that AGR2 is regulated through the interactions of multiple molecules in various pathways. How these multiple pathways are interlinked to regulate AGR2 and the associated proteins remains unaddressed. 

## 5. Role of AGR2 in the Cell Signaling Network

**Figure 3:**
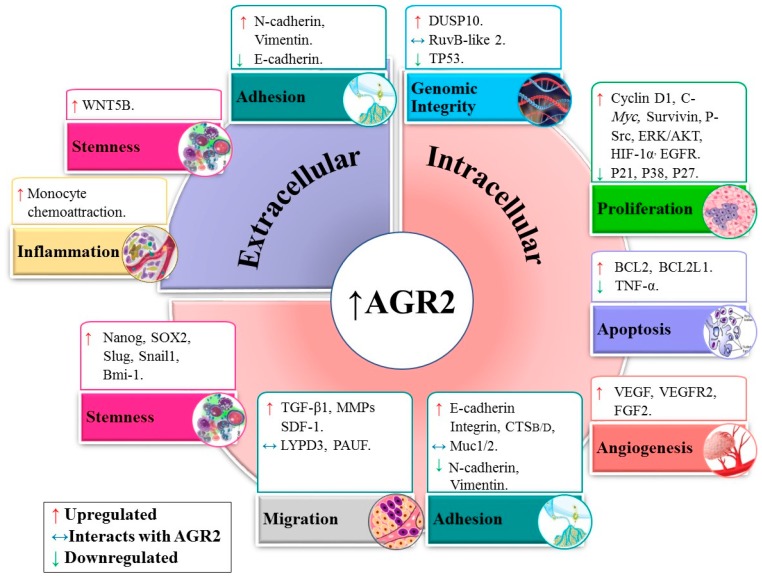
The overexpression of intracellular AGR2 and the presence of extracellular AGR2 (eAGR2) impact several pathways. AGR2 overexpression has been shown to effect pathways related to genomic integrity, apoptosis, angiogenesis, adhesion, migration, and stemness. Several proteins have been shown to be upregulated or downregulated in the presence of AGR2. In addition, several proteins have been shown to directly interact with AGR2 including DAG1, GRp78, RuvB-like 2, Muc1/2, LYPD3, and PAUF (also known as ZG16B). In addition, eAGR2 particularly affects the expression of molecules related to adhesion and stemness and is able to selectively promote monocyte attraction.

The AGR2 protein is associated with different pathways in normal and tumor cells, as shown in Figure 3. 

### 5.1. Genomic Integrity

Several studies associated the expression of AGR2 with proteins that are components of the genomic integrity pathway. In the biopsies from squamous and Barrett’s epithelium patients, analysis using immunochemical methods, electrophoresis and DNA sequencing revealed an upregulated level of AGR2, which negatively affected the genome guardian p53 and inhibited its associated pathways [8]. In a different study, the inhibition of the AGR2 protein in the prostate cancer cell lines PC-3, DU145 and LNCaP was shown to upregulate p53 [15]. The AGR2 protein was also shown to promote p53 inhibition in HCT116 cells [21]. In the lung carcinoma A540 cell line, the inhibition of AGR2 using drug treatment upregulated the p53 activity by inhibiting dual-specificity phosphatase 10 (DUSP10) [17]. Correspondingly, the knockdown of AGR2 in the breast cancer cell lines T47D and MCF7 strongly induced p53 expression [16]. AGR2 was also observed in the breast cancer cell lines MCF7 and H1299 to specifically bind to another protein called RuvB-like 2 (RUVBL2), which is involved in DNA repair, transcription regulation and chromatin structural control [84]. However, the mechanism initiated through this interaction remains to be understood. 

### 5.2. Proliferation and Apoptosis

AGR2 deregulation has been postulated to affect growth signaling pathways. The overexpression of AGR2 in the human ovarian cancer cell line MDAH-2774 enhanced cell proliferation and invasion [85]. AGR2 was overexpressed in tubular complexes and in early pancreatic intraepithelial neoplasia cells, and the deletion of the protein resulted in the inhibition of the development of pre-neoplastic lesions [86]. In GBM cell lines (T98G, A172, U87 and U251), the knockdown of AGR2 induced the cell cycle arrest in the G0/G1 phase and suppressed the migration and invasion of the U87 and U251 cells [25]. Furthermore, the overexpression of AGR2 in adenocarcinomas promoted growth and aggressiveness in an ER localization-dependent manner [87].

The AGR2-linked enhancement of cell growth has been associated with several proteins. In the breast cancer cell line T47D, silenced AGR2 protein was shown to downregulate cyclin D1, c-Myc and survivin, which play a critical role in cell growth. By contrast, recombinant AGR2 (rh AGR2) was found to upregulate cyclin D1 [88]. Similarly, the knockdown of AGR2 in the human breast cancer cell lines ZR-75-1 and T47D using siRNA resulted in the downregulation of survivin (BIRC5), c-Myc and p-Src proteins [88]. Moreover, the knockdown of AGR2 in the breast cancer cell lines T47D and MCF7 strongly induced p27 and p21 expressions [16,17]. The inhibition of the AGR2 protein in the prostate cancer cell lines PC-3, DU145 and LNCaP upregulated P21 expression [15]. A series of experiments was performed to examine the relationship between AGR2 and the ERK/AKT pathways in pancreatic cancer cells (PCa). The treatment using the fibroblast growth factor 2 (FGF2) to antagonize ERK/AKT, or the addition of AGR2 siRNA, or the combination of both, showed that silencing AGR2 inhibited the ERK/AKT pathway [63]. 

In relation to apoptosis, the knockdown of intracellular AGR2 in the high-grade HNSCC cell line CAL-27 induced apoptosis, reduced sphere formation and downregulated the anti-apoptotic proteins Bcl2 and Bcl2l1 [70]. By contrast, the secreted AGR2 protein from prostate primary cancer cells 10-076 CP and the adenocarcinoma cells LuCaP was shown to induce apoptosis in normal prostate stromal cells [73]. Apparently, these two effects of AGR2 may at first appear to be contradictory, but they can indicate the dual role of AGR2, which is likely to be dependent on the studied AGR2 isoform.

### 5.3. Angiogenesis

The AGR2 protein was shown to enhance the activities of vascular endothelial growth factor (VEGF) and FGF2, factors that promote angiogenesis, vascular endothelial cells and fibroblast invasion [89]. In the prostate cancer cell line PC-3, secreted AGR2 was shown to enhance vascular endothelial growth factor receptor (VEGFR) activity through the formation of disulphide bonds [90]. Therefore, AGR2 is likely to have an indirect effect on angiogenesis.

### 5.4. Adhesion and Migration

AGR2 has been implicated in metastatic and invasive pathways in several biological models, and its elevated expression has been associated with increased cell adhesion in several cell lines. The treatment of the ovarian cancer cell line SK-OV-3 with a DNA methyltransferase inhibitor to increase the level of AGR2 expression resulted in increased cell migration and invasion [91]. Congruently, the knockdown of the AGR2 protein using siRNAs or clustered regularly interspaced short palindromic repeat technology in several cell lines, namely, the lung adenocarcinoma cell lines A549 and H1299, the estrogen receptor-positive breast cancer cell lines BT-474 and MCF7, the pancreatic cancer cell line PANC-1 and the embryonic kidney epithelial cell line HEK-293, resulted in the decreased ability of the cancer cells to adhere [79]. 

Several experiments have shown effects of the intracellular AGR2 expression on adhesive and metastatic related molecules. In PDAC cells, intracellular AGR2 was shown to be an important posttranscriptional regulator of cathepsins B (CTSB) and D (CTSD), as both proteins were found to be upregulated in AGR2 overexpressed tumors [92]. In a study on prostate cancer cells, silencing the AGR2 protein in the PC-3 cell line resulted in the significant reduction of cellular attachment to fibronectin, collagen I, collagen IV, laminin I and fibrinogen and was associated with the sharp decrease in the expressions of α4, α5, αV, β3 and β4 integrins [93]. The overexpression of intracellular AGR2 in HEK-293 and H1299 cell lines decreased both vimentin and N-cadherin, and the downregulation of AGR2 in A549 and PANC-1 cells decreased the level E-cadherin and upregulated both vimentin and N-cadherin [79]. The AGR2 protein was shown to interact with MUC1 and MUC2 [34,80,94], which are O-glycosylated and play essential roles in forming protective mucous barriers on epithelial surfaces. In particular, the AGR2 expression was shown to be essential for the expression of MUC1 in the COLO-357 and BxPC3 cell lines [80]. Recent reviews implicate the mucin family proteins in the metastatic process by blocking apoptosis and promoting invasion, proliferation and migration in epithelial cancers [95,96]. Interestingly, the induction of the AGR2 protein in the PDAC tumor cells regulated the expression of several ER chaperones (PDI, CALU and RCN1) [92].

eAGR2 was also shown to affect adhesive and metastatic related molecules. In gastric signet-ring cell carcinoma cells, the eAGR2 protein was shown to be highly expressed extracellularly and was associated with the ability to activate stromal fibroblasts, promote cancer cells invasion and increase both the growth and the resistance to oxidative and hypoxic stresses [97]. In the metastatic cholangiocarcinoma KKU-213L5 cell line, the depletion of the AGR2vH isoform using siRNA resulted in the significant decrease in cell adhesion and in vimentin expression [98]. In lung cancer cells, AGR2 was shown to play a role in the preneoplastic phenotype, thus contributing to epithelial tumorigenicity and promoting the acquisition of invasive and metastatic features [99]. In this particular study, eAGR2 was shown to disrupt the epithelial cell–cell adhesion in non-tumorigenic normal human bronchial epithelial cells (HBEC) organoids. In addition, the expressions of 84 epithelial–mesenchymal transition (EMT) genes were quantified using the reverse transcription–quantitative reverse transcription array for HBEC organoids grown with/without eAGR2 for 10 days. As a result, matrix metalloproteinases, vimentin and N-cadherin had an increased expression, whereas E-cadherin had a decreased expression. Of note, this effect is different to that observed for the intracellular AGR2, which is consistent with an isoform-dependent function of AGR2 [12].

Other metastatic-related molecules have been associated with AGR2. Earlier works indicated that intracellular AGR2 partnered with the Ly6/PLAUR domain-containing protein 3 (LYPD3), a protein involved in cell interactions with the extracellular matrix, the provision of cancer cell mobility and metastasis [3]. In the salivary adenoid cystic carcinoma cell lines SACC-83 and SACC-LM, the knockdown of intracellular AGR2 using shRNA resulted in the reduction of TGF-β1 [70]. Through a network-based analysis, the intracellular AGR2 protein was identified to accompany the PAUF protein, which is involved in tumor metastasis, in cervical cancer cells [100]. In the GBM cell lines T98G, A172, U87 and U251, the intracellular AGR2 protein was shown to be overexpressed, and the depletion of this AGR2 reversed the action of the stromal-derived factor-1 (SDF-1), which is known to stimulate the upregulation of the EMT markers [25].

In conclusion, the deregulation of AGR2 affects the metastatic and invasive pathways. The variety of metastatic-related molecules shown to be affected by the deregulation of AGR2 may indicate a general signal transduction mechanism driven by the role of AGR2 in the UPR and proteolytic degradation.

### 5.5. Stemness

A few studies have associated AGR2 expression with stemness. The intracellular AGR2 expression was correlated with Aldehyde Dehydrogenase 1 (ALDH1), POU class 5 homeobox 1 (Oct4), SRY-box transcription factor 2 (Sox2) and snail family transcriptional repressor 2 (Slug) in the human HNSCC tissue array [70]. The overexpression of intracellular AGR2 was also observed in CSC derived from human pancreatic patient-derived xenografts [66,86]. In meningioma, the intracellular AGR2 expression was shown to co-localize with Bmi-1 and was regionally correlated with cancer stem cell markers Nestin, prominin 1 (CD133) and Sox2 in high-grade tumors [23]. In addition, AGR2-Bmi-1-positive cells had a low level of nuclear caspase-3. Two knockdown experiments supported the role of AGR2 in stemness. Homozygous AGR2−/− mice had enlarged stomachs and died prematurely, as the loss of AGR2 expression seemed to result in halting the process of epithelial cell proliferation and differentiation [33]. Correspondingly, the knockdown of AGR2 in the HNSCC cell line CAL27 reduced sphere formation and effectively decreased the CSC markers Snail family transcriptional repressor 1 (Snail1), Slug, Sox2, NANOG and OCT4 [70]. In addition growing HBEC organoids with eAGR2 for 10 days was shown to increase the expression of Wnt family member 5B (Wnt5b) [99].

## 6. AGR2 Functions through the UPR Machinery

**Figure 4:**
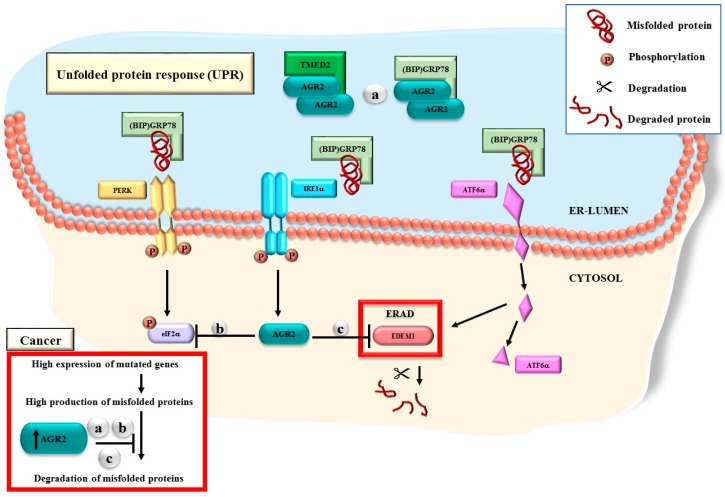
The mechanism of action for AGR2 in the UPR machinery. AGR2 has several roles in the UPR. (**a**) Dimeric AGR2 directly interact with GRp78 or TMED2 in the ER-lumen. (**b**) The AGR2 overexpression affects the expression of phosphorylated eIF2α. (**c**) The expression levels of AGR2 affects the ERAD process through the regulation of EDEM1. In cancer cells, AGR2 is likely to play a master role in preventing the degradation of mutated or misfolded proteins, thus allowing errors to be unaccounted for.

Given the ability of AGR2 to affect multiple pathways in diverse biological models, it must be able to function through a biological housekeeping machinery. Evidence collected over two decades strongly implicates the association of AGR2 with the UPR, which operates in the ER, as shown in Figure 4. Early molecular functional studies revealed the ability of the AGR2 protein to interact with α dystroglycan1 (DAG1), a membrane-bound protein postulated to play a role in the calcium uptake of the ER [3]. Landmark experiments showed that the overexpression of AGR2 was associated with induced stress in the ER [34] and that AGR2 was shown to be located in the ER vicinity and to have a fundamental role in the homoeostasis of the secretory pathway [101]. Another evidence came from the results showing that the dimerization of AGR2 protein is necessary for its interaction with other ER proteins, including the ER luminal calcium-binding protein GRp78, which is also known as heat-shock protein A5 (HSPA5) [20]. Interactions with GRp78 confirmed the role of AGR2 in the cellular responses to various stress exposures. Recently, the binding of TMED2 to dimeric AGR2 was shown to exert a selective regulation on protein folding and trafficking, and contribute to protein quality control in the ER [12]. In this study, 71 molecules were predicted to influence this process and potentially act either as activators or inhibitors of AGR2.

## 7. Drug Resistance

The AGR2 protein was shown to affect drug resistance in cancer cells that can resist radiotherapy and chemotherapy. The examination of the AGR2 protein levels in 61 breast carcinomas patients by IHC revealed their overexpression, which was associated with lower tamoxifen treatment efficacy [102]. A study showed that the AGR2 protein expression level in the samples from breast cancer patients, who were previously treated with tamoxifen, was higher than that in those who had not been treated with tamoxifen [103]. The analysis of the samples of 94 breast cancer patients using immunoblotting revealed that the AKT inhibitors had the ability to reduce the basal AGR2 levels and attenuate the induction of AGR2 by tamoxifen, which was more pronounced in cells that were treated with the combination of tamoxifen and AKT inhibitors [74]. Accordingly, AGR2 overexpression was found to be associated with chemoresistance in the human breast cancer cell lines MCF7 and MDA-MB23 [104]. Using Western blot analysis, immunofluorescence and RT-PCR, the knockdown of AGR2 was shown to suppress hypoxia-inducible factor-1α (HIF-1α)-induced doxorubicin resistance, and the increased levels of AGR2 resulted in resistance enhancement [104]. Consistently, the knockdown of AGR2 in the MCF7 and T47D breast cancer cell lines reduced the fulvestrant treatment resistance in both cell lines, whereas the overexpression of AGR2 in the MCF7 cells increased cell survival under the fulvestrant treatment [16].

The association of AGR2 with drug resistance was also studied in other cancers. The silencing of AGR2 in the MPanc-96 pancreatic cancer cell line using siRNA and shRNA significantly increased the effectiveness of gemcitabine treatments in 98% of neoplastic cells in comparison with cells that had a high AGR2 expression level [46]. The knockdown of AGR2 in six biliary tract cancer cell lines using shRNA resulted in a decreased growth by 98% and an increase in the sensitivity of the cells to chemotherapeutic drugs [68]. Similarly, the silencing of AGR2 using the overexpression of miR-217 in the CML cell line K562, which is dasatinib resistant, restored the cells’ sensitivity to dasatinib treatment [82]. In the human hepatocarcinoma cell line, both the AGR2 mRNA and protein levels were increased significantly by the treatment with the genotoxic agent 2,3,7,8-tetrachlorodibenzo-*p*-dioxin (TCDD), and this effect was mediated by the aryl hydrocarbon receptor (AhR). Of note, the phosphorylation and acetylation of p53 were inhibited in TCDD-treated cells [14]. In meningioma tissues and primary cell lines, the AGR2 expression levels and the associated effects on the chemotherapeutic agents, cisplatin and etoposide were investigated using transcriptome microarray analysis and immunofluorescence staining. The results showed that aggressive meningioma tumors had a significantly higher AGR2 protein expression and that cells that express high levels of AGR2 were more resilient to chemotherapeutic treatment [23]. The ability of the intercellular AGR2 to enhance drug resistance is likely to be consequential to its ability to regulate the ERAD pathway, while expression of eAGR2 is likely to contribute to the resilience of cancer cells that are surviving in an inflammatory microenvironment. Future experimental work is needed to determine the precise mechanisms utilized by AGR2 to enhance drug resistance, and uncover associated drug sensitizing methods.

## 8. Therapeutic Potential

Antibodies and peptides have been developed to target AGR2. Using bead-based and flow cytometry, a DNA aptamer C14B1 was identified for its G-rich G-quadruple region and was found to specifically bind to AGR2 [105]. In breast cancer cells, an antibody (18A4 mAb) that showed a specific binding to AGR2 in the cytoplasm using immunofluorescent staining was revealed to inhibit the growth of the breast cancer cell line MCF7 [106]. In colorectal cancer cells, AGR2 was also shown to be a potentially useful antigenic target for cancer immunotherapy. Human colorectal cancer cell lines that have a high AGR2 expression were targeted using dendritic cells (DCs) that express the AGR2 gene (AdAGR2). The outcome showed that the AdAGR2 transduction in DCs enhanced the DC activation and maturation by inducing higher expressions of some genes, such as CD80, CD86 and HLA-DR [107]. Correspondingly, two AGR2 peptides capable of binding the leukocyte antigen (HLA)-A*0201 were generated. These peptides were utilized to generate specific cytotoxic T-lymphocytes (CTLs). DCs pulsed with the mentioned peptides were used to activate the specific CTLs in AGR2-positive and -negative colorectal cancer cell lines. Lysis of the incubated CTLs was found only in AGR2-positive cell lines. Therefore, these peptides can potentially be employed to target cancer cells that are positive for AGR2 [108]. The caveat is that AGR2 can be detected in normal colon cells [18]. In a cell migration assay, an AGR2 binding peptide (H10) was shown to be sufficient to prevent cancer cell migration in breast and prostate cancer cells [109]. The antibody-induced blocking of AGR2 in pancreatic ductal adenocarcinoma cell lines revealed a significantly reduced tumor growth and metastasis and led to tumor regression and improved survival [110]. Furthermore, humanized antibodies selected to block the AGR2 protein were shown to effectively inhibit tumor growth in a xenograft model [111]. In a recent study on CML, the eAGR2 protein was used as a vital indicator for monitoring the normalization of blood vessels during an anti-angiogenic therapy utilizing nanoparticles (AuNPs). The detected AGR2 level was affected by the AuNP treatment in tumor tissues and plasma [112]. 

## 9. AGR2 Gene Expression Meta-Analysis in CNS Tumors

AGR2 overexpression has been detected in CNS tumors, including GBM and high grade meningiomas [23,24,25]. To further explore the pathways associated with the high expression of AGR2, a meta-analysis on CNS tumors was conducted. The search terms brain tumors, human and expression were used as queries in GEO and ArrayExpress to identify the microarray expression data sets on primary brain tumors, which were utilized to extract the AGR2 expression data for further analysis [113,114].

The data sets were analyzed using the Transcriptome Analysis Console v4.0.1 (Thermo Fisher Scientific Waltham, MA, USA) that includes the LIMMA (linear modeling for microarrays) package from Bioconductor [115]. The data sets, in which the range of the AGR2 log2 signal intensity values between samples with the lowest and highest signal intensities was approximately greater than 4, were subjected to differentially expression analysis (Table 1). 

With one exception, the low AGR2 expression samples were regarded as those that had signal intensities at the lower 40% of the log2 signal intensities. The remaining samples of each data set were regarded as AGR2 high expression samples. The threshold between AGR2 low and high expression samples was empirically determined with the support of a permutation test. Notably, the adult meninges control samples (*N* = 13) and dura control samples (*N* = 3) of the GEO submission GSE84263 revealed AGR2 expression levels in the range of AGR2 low expressing meningiomas, whereas the embryonic meninges control samples (*N* = 9) revealed an expression pattern mostly in the range of the high expressing meningiomas. The over-activation of the characteristic biomarkers and crucial embryonic signaling pathways in human adamantinomatous craniopharyngiomas was described in an integrative mutational and transcriptome profiling study [116]. A previous work showed that AGR2 was highly expressed in aggressive meningioma cells [23], and a comparison of the RNA expression levels of AGR2 published by an independent study [117], showed a significantly higher average expression level in grade II+ tumors or tumors that recurred, than in grade I tumors (Grade I:104.285, grade II+/recurred:988.182, *T*-test *p* = 0.021).

**Figure 5:**
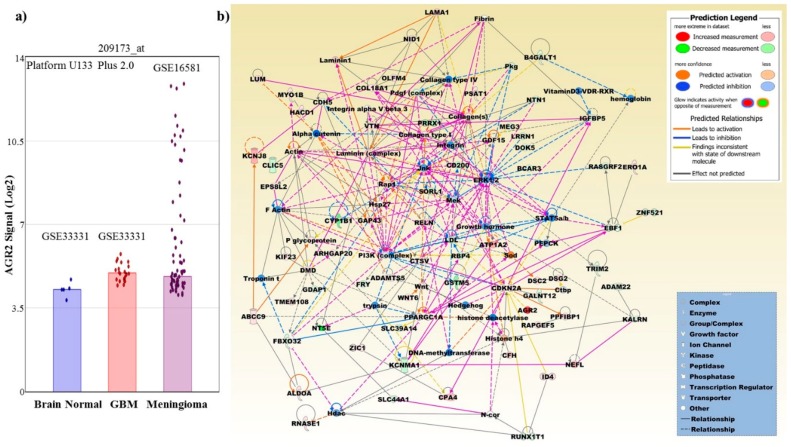
(**a**) Using HG 133 Plus 2.0 microarrays and a probe set (209173_at) interrogating mostly the coding region between exon 4 to exon 8, a significant higher expression is revealed for meningiomas compared to normal brain tissue and GBM. Normal brain and GBM files were derived from GEO submission GSE33331 and meningiomas from GSE16581. Dots indicate expression values of individual samples. Files were analyzed using Transcriptome Analysis Software (Thermo Fisher Scientific). Stars mark *p*-values < 0.05. (**b**) The merged network is based on the top three networks that were most significantly related to the microarray expression profiles of 245 DEGs that were retrieved from the meta-analysis of microarray expression data on meningiomas and were observed in at least three different studies. The Ingenuity Pathway Analysis (IPA) (Qiagen, Hilden, Germany) and its curated molecular knowledge base were employed to generate the merged network. Network molecules are related skeletal and muscular system development and function, cancer, endocrine system disorders, embryonic development, nervous system development and function, cellular function and maintenance, and tissue development (Table 2). Upregulated network molecules include, ABCC9, ADAMTS5, AGR2, ALDOA, ARHGAP20, ATP1A2, CDKN2A, CFH, COL18A1, CPA4, CTSV, DMD, DSC2, DSG2, EPS8L2, ERO1A, GAP43, GDF15, HACD1, ID4, KCNJ8, KIF23, LAMA1, LUM, MYO1B, NEFL, NID1, OLFM4, PPARGC1A, PPFIBP1, PSAT1, RAPGEF5, RELN, RNASE1, TMEM108, VTN, WNT6, and ZIC1. Downregulated network molecules include, ADAM22, B4GALT1, BCAR3, CD200, CDH5, CLIC5, CYP1B1, DOK5, EBF1, FBXO32, FRY, GALNT12, GDAP1, GSTM5, IGFBP5, KALRN, KCNMA1, LRRN1, MEG3, NT5E, NTN1, PRRX1, RASGRF2, RBP4, RUNX1T1, SLC39A14, SLC44A1, SORL1, TRIM2, and ZNF521. Molecules retrieved from the IPA molecular base include, actin, alpha catenin, collagen type I, collagen type IV, collagen(s), Ctbp, DNA-methyltransferase, ERK1/2, F Actin, fibrin, growth hormone, Hdac, hedgehog, hemoglobin, histone deacetylase, Histone h4, Hsp27, integrin, integrin alpha V beta 3, Jnk, laminin (complex), laminin1, LDL, Mek, N-cor, P glycoprotein, Pdgf (complex), PEPCK, PI3K (complex), Pkg, Rap1, Sod, STAT5a/b, troponin t, trypsin, vitaminD3-VDR-RXR, and Wnt.

We found that AGR2 was overexpressed in a subset of meningiomas, childhood craniopharyngiomas and pituitary gonadotrope tumors [118,119]. A network based on 245 differentially expressed genes (DEGs) from a comparison of AGR2 high versus low expressing meningiomas is shown in Figure 5. The 245 DEGs have an overlap of approximately 11% to the 494 annotated DEGs derived from the comparison of AGR2 high versus low expressing adCPs (GSE68015) and of approximately 26% to the 1909 annotated DEGs derived from AGR2 high versus low expressing PGTs (GSE26966) (Supplementary Table S1). Table 2 presents the predicted differential pathways generated by the Ingenuity Pathway Analysis software for meningioma tumors with a high AGR2 expression in comparison with tumors with low AGR2 expression. 

**Figure 6:**
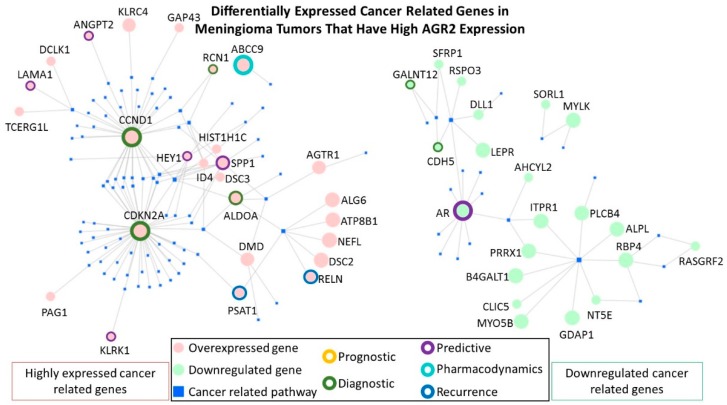
The most clinically relevant differentially expressed cancer-related genes in meningioma tumors that have high AGR2 expression.

In order to select for the most clinically relevant DEGs that are found in the list of AGR2 high versus low expressing meningiomas, the highly expressed genes or the downregulated genes were analyzed using the Network Analyst platform [120,121]. Maps were generated for the “Gene-disease Associations”, which are based on the DisGeNET database, and only cancer related pathways were included. All Network Analyst selected genes were then checked manually for clinical relevance by searching for the gene properties in “genecards.org”, searching in google for genes included in available diagnostic onco-gene panels, and by searching in “PubMed” for clinically relevant publications. Figure 6 shows the differentially expressed cancer related genes and highlights the genes that are most clinically relevant. Twelve clinically relevant overexpressed cancer-related genes were identified in AGR2 high meningiomas including: ABCC9, ALDOA, CCND1, CDKN2A, PSAT1, RCN1, RELN, ANGPT2, KLRK1, LAMA1, SPP1, and HEY1. While only three downregulated clinically relevant cancer-related genes were identified in AGR2 high meningiomas (AR, CDH5, and GALNT12).

Owing to a shortage of larger series, no concrete conclusions could be drawn regarding which subtypes of pituitary tumors are characterized by the overexpression of AGR2 levels. Although the list was not exhaustive, we did not find a significantly different overexpression of AGR2 in medulloblastomas, gliomas or glioma cells (GSE41842, GSE37418, GSE7181, GSE15824, GSE36245, GSE33331, GSE34824, GSE36245 and GSE53733) in comparison with meningiomas, adCP and GPT using the probe set (209173_at), which interrogates mostly the coding region between exon 4 to exon 8. Interestingly, a significant higher expression was revealed for GBM than for normal brain tissues when analyzing data using a probe set (228969_at), which interrogates the 3′UTR of AGR2 (brain_normal average log2 intensities: 3.87, GBM average log2 intensities: 4.76, fold change:1.86, *p* = 0.0141). The reason why this difference is observed in GBM requires further investigation. 

Detailed descriptions of the clinical relevance of the selected genes and their references were included in Table 3. 

## 10. Conclusions and Way Forward

Understanding signaling pathways at the molecular level has led to the development of promising diagnostic and prognostic biomarkers, which can help in tracking disease progression and predicting patient survival. In this review, the emerging biomarker, anterior gradient homologue 2, was meticulously evaluated on the basis of relevant and validated publications. AGR2 was shown to be highly expressed in several tumors, and its expression levels were associated with resilience to chemotherapeutic treatments. The primary protein structures of the variants for AGR2 were summarized. Moreover, the knowledge retrieved from biologically varied experimental models on the regulation of AGR2 and its associated molecules was reviewed. The top molecules implicated in regulating the expression of AGR2 included IRE1α, ATF6α, AKT, FOXA1/2, HIF-1, TMED2, and the frequently mutated tumor suppressor p53. 

The AGR2-associated molecular functions and pathways were updated. The mechanisms that could enable the rampant oncogenic effects of AGR2 were presented. AGR2 protein can affect multiple pathways through its disulphide isomerase activity, a divergent peptide substrate-binding loop, an ability to interfere with protein degradation, and an ability to release eAGR2 to induce monocyte attraction. These pathways include genomic integrity, proliferation, apoptosis, angiogenesis, adhesion, migration, stemness and inflammation. Successful antibodies and peptides were developed to target AGR2 protein that could potentially be used to target cancer cells that are positive for AGR2. 

Data from a gene expression meta-analysis considered AGR2 as an important biomarker for disease progression in meningiomas. The top associated pathways in AGR2 high CNS tumors were listed, including axonal guidance signaling and glioblastoma multiforme signaling. The top clinically relevant deregulated cancer genes in AGR2 high meningiomas were presented. However, further functional evaluations are needed to convert the application of AGR2 and the possible related molecules into clinical practice, particularly to provide effective diagnostic, prognostic and therapeutic applications that utilize the presence of AGR2 in different tumors to tip the balance toward successful outcomes.

## Figures and Tables

**Table 1:** Microarray expression studies included to investigate differentially expressed genes in high AGR2 expression tumors versus low AGR2 expression tumors.

GEO/Array Express Access Number	Array Type	Number of Samples		Tumor Type
		*AGR2* High	*AGR2* Low	
GSE54934	HuGene 1.0 ST	3	19	MN
GSE77259, GSE100534	HuGene 1.0 ST	6	16	MN
GSE88720	HuGene 2.1 ST	2	12	MN
GSE16581	U133 Plus 2.0	16	52	MN
GSE68015	U133 Plus 2.0	3	6	MN
E-MTAB-1852	U133 Plus 2.0	5	10	MN
E-GEOD-9438	U133 Plus 2.0	3	16	MN
E-MEXP-3586	U133 Plus 2.0	5	11	MN
GSE68015	U133 Plus 2.0	5	10	adCP ^1^
GSE26966	U133 Plus 2.0	13	9	PGT ^2^

^1^ adCP, adamantinomatous craniopharyngiomas; ^2^ PGT, pituitary gonadotrope tumors. Of note, different types of pituitary tumors exhibit variations in AGR2 expression.

**Table 2:** Predicted differential pathways generated by the Ingenuity Pathway Analysis Software (IPA) for meningioma tumors with high AGR2 expression compared with tumors with low AGR2 expression.

**Top Canonical Pathways**	***p*** **-Value**	**Overlap**
Apelin Cardiac Fibroblast Signaling Pathway	7.55E−05	18.2% (4/22)
Axonal Guidance Signaling	2.76E−04	3.0% (15/498)
Osteoarthritis Pathway	4.17E−04	4.3% (9/211)
Hepatic Fibrosis/Hepatic Stellate Cell Activation	8.22E−04	4.3% (8/186)
Glioblastoma Multiforme Signaling	2.21E−03	4.1% (7/170)
**Top Upstream Regulator**	***p*** **-Value of Overlap**	
progesterone	1.31E−08	
CTNNB1	1.68E−08	
ERBB2	4.47E−08	
forskolin	5.07E−08	
AHR	5.61E−08	
**Diseases and Disorders**	***p*** **-Value, # Molecules**	
Neurological Disease	2.24E−04–1.51E−15, 112	
Cancer	2.47E−04–2.22E−15, 228	
Gastrointestinal Disease	2.47E−04–2.22E−15, 216	
Organismal Injury and Abnormalities	2.47E−04–2.22E−15, 229	
Cardiovascular Disease	1.74E−04–3.10E−15, 85	
**Molecular and Cellular Functions**	***p*** **-Value, # Molecules**	
Cellular Movement	2.36E−04–9.50E−14, 97	
Cell Death and Survival	2.05E−04–1.74E−10, 97	
Cell-To-Cell Signaling and Interaction	1.95E−04–2.26E−10, 49	
Cellular Assembly and Organization	2.51E−04–2.26E−10, 70	
Cell Morphology	2.31E−04–1.15E−09, 75	
**Physiological System Development and Function**	***p*** **-Value, # Molecules**	
Organismal Development	1.87E−04–1.46E−15, 121	
Nervous System Development and Function	2.36E−04–1.51E−15, 90	
Cardiovascular System Development and Function	1.87E−04–1.60E−15, 74	
Skeletal and Muscular System Development and Function	2.31E−04–4.41E−11, 70	
Tissue Morphology	2.36E−04–9.89E−11, 97	
**Assays: Clinical Chemistry and Hematology**	***p*** **-Value, # Molecules**	
Increased Levels of Alkaline Phosphatase	6.39E−03–1.48E−03, 5	
Increased Levels of Hematocrit	1.94E−02–1.94E−02, 4	
Increased Levels of Red Blood Cells	2.43E−02–2.43E−02, 4	
Decreased Levels of Albumin	6.16E−02–6.16E−02, 1	
Increased Levels of ALT	8.12E−02–8.12E−02, 1	
**Cardiotoxicity**	***p*** **-Value, # Molecules**	
Cardiac Dysfunction	2.65E−01–2.23E−10, 19	
Cardiac Arteriopathy	1.56E−01–3.78E−08, 19	
Cardiac Arrythmia	3.92E−01–3.97E−07, 13	
Cardiac Enlargement	1.19E−01–3.81E−06, 23	
Cardiac Dilation	2.49E−01–1.25E−05, 15	
**Hepatotoxicity**	***p*** **-Value, # Molecules**	
Liver Hyperplasia/Hyperproliferation	5.24E−01–2.28E−07, 118	
Liver Steatosis	1.91E−01–1.14E−04, 14	
Liver Cirrhosis	6.16E−02–2.42E−04, 12	
Hepatocellular Carcinoma	3.86E−01–3.32E−04, 27	
Liver Necrosis/Cell Death	2.94E−02–1.23E−03, 9	
**Nephrotoxicity**	***p*** **-Value, # Molecules**	
Renal Damage	3.92E−01–4.38E−05, 12	
Renal Dysfunction	3.29E−04–3.29E−04, 2	
Renal Necrosis/Cell Death	2.65E−01–1.69E−03, 13	
Glomerular Injury	3.07E−01–9.78E−03, 8	
Renal Inflammation	3.78E−01–1.25E−02, 7	
**Top Networks**	**Score**	
Skeletal and Muscular System Development and Function, Cancer, Endocrine System Disorders	43	
Skeletal and Muscular System Development and Function, Embryonic Development, Nervous System Development and Function	38	
Cellular Function and Maintenance, Skeletal and Muscular System, Development and Function, Tissue Development	38	
Cardiovascular Disease, Cardiovascular System. Development andFunction, Organismal Injury and Abnormalities	31	
Organismal Development, Organismal Functions, Cardiac Dysfunction	29	
**Top Tox Lists**	***p*** **-Value**	**Overlap**
Hepatic Fibrosis	1.67E−05	7.5% (8/106)
Cardiac Necrosis/Cell Death	1.73E−05	4.4% (13/297)
Cardiac Fibrosis	2.61E−05	4.9% (11/223)
Recovery from Ischemic Acute Renal Failure (Rat)	3.85E−04	21.4% (3/14)
Cardiac Hypertrophy	4.86E−04	3.3% (12/362)
**Fold Change Up-Regulated Top Molecules, Exp. Value**	**Fold Change Down-Regulated Top Molecules, Exp. Value**	
AGR2, 90.210	TCEAL2, −48.790	
CALB1, 49.780	COL8A1, −36.360	
KCNT2, 34.470	MFAP5, −35.660	
DSC3, 33.800	NT5E, −31.300	
HOPX, 27.670	SYNPO2, −29.750	
GPR83, 22.020	SLPI, −20.350	
CA3, 21.830	CYP1B1, −18.420	
ATP1A2, 21.780	RSPO3, −17.040	
KCNJ8, 19.690	SFRP1, −16.210	
GRB14, 17.690	UCHL1, −15.030	

**Table 3:** Clinically relevant deregulated cancer-related genes identified in AGR2 high meningiomas.

Gene	Clinical Relevance
**Upregulated**	
ABCC9 (also known as SUR2)	The ATP Binding Cassette Subfamily C Member 9 gene encodes a protein which is a member of the superfamily of ATP-binding cassette (ABC) transporters and is known to be involved in multi-drug resistance, particularly in human cervical cancer [122].
ALDOA	The aldolase, fructose-bisphosphate A gene encodes a proteins, which belongs to the class I fructose-bisphosphate aldolase protein family. This protein is thought to catalyze the reversible conversion of fructose-1,6-bisphosphate to glyceraldehyde 3-phosphate and dihydroxyacetone phosphate, a process which is vital for developmental pathways. The elevated protein levels were shown to highly correlate with a poor prognosis in patients with non-small cell lung cancer (NSCLC) [123].
CCND1	Cyclin D1 protein encoded by the CCND1 gene belongs to the cyclin family that consists of proteins that functions as regulators of CDK kinases. The CCND1 cyclin was shown to be required for the cell cycle G1/S transition, and associated gene overexpression has been observed frequently in a variety of tumors [124]. Previous studies confirmed the downregulation of cyclin D1 upon silencing of AGR2, as mentioned in Figure 3 [88].
CDKN2A	The Cyclin Dependent Kinase Inhibitor 2A gene encodes the two cell cycle regulators p14ARF and p16INK4a. Overexpression of p16INK4a has been demonstrated in benign tumors and is seemingly a negative response mechanism to oncogenic signaling that otherwise would enhance cell proliferation [125]. In contrast, in cancers with retinoblastoma (RB) inactivation, overexpression of p16INK4a is associated with worse prognosis and therefore, using p16INK4a as a biomarker should be carefully considered in the specific context. A meta-analysis on microarray expression data in meningiomas identified a data set of 18 genes including CDKN2 whose deregulated expression was a predictive marker for recurrence [126]. This gene is often included in diagnostic onco-gene panels.
PSAT1	Phosphoserine Aminotransferase 1 encodes an enzymatic component of the serine synthesis pathway. Overexpression of PSAT1 in different cancer types is associated with negative prognosis [127]. In glioblastoma cells, the multi-kinase inhibitor regorafenib effected a lethal autophagy arrest by stabilizing PSAT1 [128].
RCN1	A class prediction analysis demonstrated that Reticulocalbin 1, a calcium-binding protein, is overexpressed in meningiomas with cytogenetically complex karyotypes that are commonly associated with unfavorable prognosis [129]. AGR2 has been shown to regulate the expression of this ER chaperone in the PDAC tumor cells [92].
RELN	The Reelin gene encodes an extracellular matrix glycoprotein protein which is involved in cell-cell interactions and is critical for cell positioning and neuronal migration during brain development. The expression of the RELN gene has been shown to be increased in Her2+ breast cancer brain metastases [130], and to be negatively correlated with the overall survival in multiple myeloma patients [131].
ANGPT2	A meta-analysis on microarray expression data in meningiomas identified a data set of 18 genes including Angiopoietin 2 whose deregulated expression was a predictive marker for recurrence [126]. Serum ANGPT2 is currently assessed for its application as a predictive and prognostic marker for immune checkpoint therapy in cancer [132].
KLRK1	Killer Cell Lectin Like Receptor K1 encodes the transmembrane protein NKG2D, which is part of the CD94/NKG2 family of C-type lectin-like transmembrane proteins. This receptor constitutes a possible therapeutic target for immune diseases and cancer [133]. In meningiomas, a microarray expression analysis detected higher expression of the read-through transcript KLRK1-KLRC4 in female cases compared male cases [134].
LAMA1	Laminin Subunit Alpha 1 encodes for a protein, which is a component of the extracellular matrix and was found to be comparably higher expressed in breast cancer stem cells [135]. A meta-analysis on microarray expression data on cancer stem cells in meningiomas identified recurrently upregulation of LAMA1 in GII + GIII compared to GI meningiomas [136].
SPP1	Secreted phosphoprotein 1 (SPP1) gene encodes a protein which is involved in the attachment of osteoclasts to the mineralized bone matrix, and in the upregulated expression of interferon-gamma and interleukin-12. The protein overexpression has been observed in various malignant neoplasms including medullary thyroid carcinoma, lung cancer, gastric cancer, breast cancer and colorectal cancer [137]. In addition, the upregulation of SPP1 protein has been shown to be significantly associated with adherence and invasion [138].
HEY1	The hes related family bHLH transcription factor with YRPW motif 1 (HEY1) gene encodes a nuclear protein that belongs to the hairy and enhancer of split-related (HESR) family of transcriptional repressors. Notch and c-Jun signal transduction pathways has been shown to induce the expression of this gene. HEY1 expression has been shown to increase with increasing astrocytoma tumor grade and to correlate with decreased overall survival and disease-free survival [139]. However, it is important to note that the protein levels of HEY1 have been reported to be high in the normal brain [37].
**Downregulated**	
AR	The Androgen Receptor gene encodes a protein that functions as a steroid-hormone activated transcription factor. Many studies have shown tumor associated overexpression of this gene [140]. However, in meningioma, our data shows a significant downregulation of AR expression. Other studies have linked low expression with poorer prognosis. The loss of the AR expression in triple-negative breast cancers was associated with a worse prognosis [141]. Loss of AR expression was also shown to promote a stem-like cell phenotype and progression in prostate cancer cells through the STAT3 signaling pathway [142]. In addition, a double conditional knockout of adenomatous polyposis coli and Smad4 caused invasive prostate cancers that had lost the expression of the AR [143].
CDH5	The cadherin 5 gene encodes for a glycoprotein important for cell-cell adhesion. The protein is thought to contribute to endothelial cell biology by organizing intracellular junctions. The high expression of CDH5 protein, also known as vascular endothelial (VE-) cadherin, has been strongly associated with aggressiveness in many tumors, including breast cancer, melanoma, and small cell lung cancer [144]. However, deficiency of VE-cadherin was also observed in tumors, such as angiosarcomas [145]. Furthermore, absence of VE-cadherin expression has been associated with epithelial mesenchymal transition (EMT) [146]. Perhaps lower expression of VE-cadherin in meningiomas with high AGR2 promotes a smoother EMT.
GALNT12	The polypeptide N-acetylgalactosaminyltransferase 12 gene encodes an enzyme which catalyzes the modulation of N-acetylgalactosamine (GalNAc) on a polypeptide acceptor as part of O-linked protein glycosylation. Gene loss and protein defects have been strongly associated with susceptibility to Colorectal Cancer 1 and Familial Colorectal Cancer Type X [147]. Perhaps the downregulation of GALNT12 is indicative of deregulated glycosylation pathways in meningioma.

## Supplementary Materials

The following are available online at https://www.mdpi.com/2072-6694/11/7/890/s1, Table S1: All 245 differentially expressed genes detected in meningiomas with high AGR2 expression.

## Abbreviations

AdAGR2	Encode AGR2
AdCs	Adenocarcinoma
AGR2	Anterior Gradient 2 Homolog
AhR	Aryl hydrocarbon receptor
AKT1	RAC-alpha serine/threonine-protein kinase1
ALDH1	Aldehyde Dehydrogenase 1
ATF6α	Activating Transcription Factor 6
AuNPs	Nanoparticles
CALB1	Calbindin 1
CNS	Central Nervous System tumors
CCA	Cholangiocarcinoma
CCA	Cholangiocarcinoma
CD133	Prominin 1
CDH2	N-Cadherin
CDH1	E-Cadherin
CML	Chronic Myelogenous Leukemia
CRISPR	Clustered Regularly Interspaced Short Palindromic Repeats
CSCs	Cancer Stem Cells
CTLs	Cytotoxic T-lymphocyte
CTSB	Cathepsins B
CTSD	Cathepsins D
DAG1	α dystroglycan1
DCs	Dendritic Cells
DFS	Disease free survival
DUSP10	Dual Specificity Phosphatase 10
eAGR2	extracellular AGR2
EBP1	ErbB3 Binding Protein 1
EDEM1	Alpha-mannosidase like protein1
EGFR	Epidermal Growth Factor Receptor
eIF2α	Translation Initiation Factor
ELISA	Enzyme-Linked Immunosorbent Assay
EMT	Epithelial-mesenchymal transition
ER	Endoplasmic Reticulum
ERAD	Endoplasmic Reticulum-Associated Degradation
FGF2	Fibroblast Growth Factor 2
FOXA1	Forkhead Box A1
FOXA2	Forkhead Box A3
FOXM1	Forkhead Box M1
GBM	Glioblastoma Multiforme
GEO	Gene Expression Omnibus
GEP	Gene Expression Profiling
GRp78	Endoplasmic reticulum luminal Calcium Binding Protein
H&E	Hematoxylin and eosin stain
HBEC	Human Bronchial Epithelial Cells
HCCs	Hepatocellular carcinoma
HepG2	Human Hepatocarcinoma cell line
HER2	Human Epidermal Growth Factor Receptor2
HIF-1α	Hypoxia-inducible factor-1α
HNSCC	Head and Neck Squamous Cell Carcinoma
IBD	Inflammatory Bowel Disease
IDCs	Invasive Ductal Carcinomas
IPA	Ingenuity Pathway Analysis Software
IRE1α	Inositol-Requiring Enzyme1
K562DR	Dasatinib-resistant K562
LYPD3	Ly6/PLAUR domain-containing protein 3
MAPK	Mitogen Activated Kinase-Like Protein
miR-342-3p	MicroRNA-342-3p
MMPs	Matrix metalloproteinases
mRNA	Messenger RNA
MUC1	Mucin1
miR-1291	MicroRNA-1291
NSCLC	Non-small cell lung cancer
Oct4	POU class 5 homeobox 1
OS	Overall Survival
PA	Pituitary Adenoma
PanINs	Pancreatic Intraepithelial Neoplasia
PCa	Pancreatic cancer cell line
PC-3	Prostate cancer cell line
PDAC	Pancreatic Ductal Adenocarcinomas
PDI	Protein Disulfide Isomerase
PDPK1	3-phosphoinositide-dependent protein kinase 1
PIMA	Pulmonary Invasive Mucinous Adenocarcinoma
PR	Progesterone Receptor
RAD9A	RAD9 checkpoint clamp component A
rhAGR2	Recombinant AGR2
RNA-seq	Transcript Profiling
RT-PCR	Reverse transcription
RT-qPCR	Reverse transcription-Quantitative reverse transcription
RuvB-like2	Reptin
SACC	Salivary Adenoid Cystic Carcinoma cell line
SCCs	Squamous Cell Carcinoma
SDF-1	Stromal-Derived Factor-1
shRNA	Short Hairpin RNA
siRNA	Small interferon RNA
Slug	Snail family transcriptional repressor 2
Snail1	Snail family transcriptional repressor 1
Sox2	SRY-box transcription factor 2
SP	Secretory Pathway
SRCC	Gastric Signet-Ring Cell Carcinoma
TC	Tubular Complexes
TCDD	2,3,7,8-Tetrachlorodibenzo-p-dioxin
TMED2	Transmembrane p24 trafficking protein 2
TGF-β	Transforming Growth Factor Beta
TTP	Time to Tumor Progression
TKI	Tyrosine Kinase Inhibitor
TNBC	Triple-negative breast cancers
TNF-α	Tumor Necrosis Factor
TNM	Tumor node metastasis
UPR	Unfolded Protein Response
VEGF	Vascular Endothelial Growth Factor
VEGFR2	Vascular Endothelial Growth Factor Receptor
Wnt5b	Wnt family member 5B

## References

[1] Higa A., Mulot A., Delom F., Bouchecareilh M., Nguyen D.T., Boismenu D., Wise M.J., Chevet E. (2011). Role of pro-oncogenic protein disulfide isomerase (pdi) family member anterior gradient 2 (agr2) in the control of endoplasmic reticulum homeostasis. J. Biol. Chem..

[2] Thompson D.A., Weigel R.J. (1998). Hag-2, the human homologue of the xenopus laevis cement gland gene xag-2, is coexpressed with estrogen receptor in breast cancer cell lines. Biochem. Biophys. Res. Commun..

[3] Fletcher G.C., Patel S., Tyson K., Adam P.J., Schenker M., Loader J.A., Daviet L., Legrain P., Parekh R., Harris A.L. (2003). Hag-2 and hag-3, human homologues of genes involved in differentiation, are associated with oestrogen receptor-positive breast tumours and interact with metastasis gene c4.4a and dystroglycan. Br. J. Cancer.

[4] Liu D., Rudland P.S., Sibson D.R., Platt-Higgins A., Barraclough R. (2005). Human homologue of cement gland protein, a novel metastasis inducer associated with breast carcinomas. Cancer Res..

[5] Patel P., Clarke C., Barraclough D.L., Jowitt T.A., Rudland P.S., Barraclough R., Lian L.Y. (2013). Metastasis-promoting anterior gradient 2 protein has a dimeric thioredoxin fold structure and a role in cell adhesion. J. Mol. Biol..

[6] Kumar A., Godwin J.W., Gates P.B., Garza-Garcia A.A., Brockes J.P. (2007). Molecular basis for the nerve dependence of limb regeneration in an adult vertebrate. Science.

[7] Gourevitch D.L., Clark L., Bedelbaeva K., Leferovich J., Heber-Katz E. (2009). Dynamic changes after murine digit amputation: The mrl mouse digit shows waves of tissue remodeling, growth, and apoptosis. Wound Repair Regen..

[8] Pohler E., Craig A.L., Cotton J., Lawrie L., Dillon J.F., Ross P., Kernohan N., Hupp T.R. (2004). The barrett’s antigen anterior gradient-2 silences the p53 transcriptional response to DNA damage. Mol. Cell. Proteom..

[9] Gray T.A., Alsamman K., Murray E., Sims A.H., Hupp T.R. (2014). Engineering a synthetic cell panel to identify signalling components reprogrammed by the cell growth regulator anterior gradient-2. Mol. Biosyst..

[10] Niederreiter L., Kaser A. (2011). Endoplasmic reticulum stress and inflammatory bowel disease. Acta Gastro-Enterol. Belg..

[11] Zhu Q., Mangukiya H.B., Mashausi D.S., Guo H., Negi H., Merugu S.B., Wu Z., Li D. (2017). Anterior gradient 2 is induced in cutaneous wound and promotes wound healing through its adhesion domain. FEBS J..

[12] Maurel M., Obacz J., Avril T., Ding Y.P., Papadodima O., Treton X., Daniel F., Pilalis E., Horberg J., Hou W. (2019). Control of anterior gradient 2 (agr2) dimerization links endoplasmic reticulum proteostasis to inflammation. EMBO Mol. Med..

[13] Zweitzig D.R., Smirnov D.A., Connelly M.C., Terstappen L.W., O’Hara S.M., Moran E. (2007). Physiological stress induces the metastasis marker agr2 in breast cancer cells. Mol. Cell. Biochem..

[14] Ambolet-Camoit A., Bui L.C., Pierre S., Chevallier A., Marchand A., Coumoul X., Garlatti M., Andreau K., Barouki R., Aggerbeck M. (2010). 2,3,7,8-tetrachlorodibenzo-p-dioxin counteracts the p53 response to a genotoxicant by upregulating expression of the metastasis marker agr2 in the hepatocarcinoma cell line hepg2. Toxicol. Sci..

[15] Hu Z., Gu Y., Han B., Zhang J., Li Z., Tian K., Young C.Y., Yuan H. (2012). Knockdown of agr2 induces cellular senescence in prostate cancer cells. Carcinogenesis.

[16] Li Z., Zhu Q., Chen H., Hu L., Negi H., Zheng Y., Ahmed Y., Wu Z., Li D. (2016). Binding of anterior gradient 2 and estrogen receptor-alpha: Dual critical roles in enhancing fulvestrant resistance and igf-1-induced tumorigenesis of breast cancer. Cancer Lett..

[17] Hrstka R., Bouchalova P., Michalova E., Matoulkova E., Muller P., Coates P.J., Vojtesek B. (2016). Agr2 oncoprotein inhibits p38 mapk and p53 activation through a dusp10-mediated regulatory pathway. Mol. Oncol..

[18] Brychtova V., Mohtar A., Vojtesek B., Hupp T.R. (2015). Mechanisms of anterior gradient-2 regulation and function in cancer. Semin. Cancer Biol..

[19] Chevet E., Fessart D., Delom F., Mulot A., Vojtesek B., Hrstka R., Murray E., Gray T., Hupp T. (2013). Emerging roles for the pro-oncogenic anterior gradient-2 in cancer development. Oncogene.

[20] Ryu J., Park S.G., Lee P.Y., Cho S., Lee D.H., Kim G.H., Kim J.H., Park B.C. (2013). Dimerization of pro-oncogenic protein anterior gradient 2 is required for the interaction with bip/grp78. Biochem. Biophys. Res. Commun..

[21] Clarke D.J., Murray E., Faktor J., Mohtar A., Vojtesek B., MacKay C.L., Smith P.L., Hupp T.R. (2016). Mass spectrometry analysis of the oxidation states of the pro-oncogenic protein anterior gradient-2 reveals covalent dimerization via an intermolecular disulphide bond. Biochim. Biophys. Acta.

[22] Tian S.B., Tao K.X., Hu J., Liu Z.B., Ding X.L., Chu Y.N., Cui J.Y., Shuai X.M., Gao J.B., Cai K.L. (2017). The prognostic value of agr2 expression in solid tumours: A systematic review and meta-analysis. Sci. Rep..

[23] Khan I., Baeesa S., Bangash M., Schulten H.J., Alghamdi F., Qashqari H., Madkhali N., Carracedo A., Saka M., Jamal A. (2017). Pleomorphism and drug resistant cancer stem cells are characteristic of aggressive primary meningioma cell lines. Cancer Cell Int..

[24] Hong X.Y., Wang J., Li Z. (2013). Agr2 expression is regulated by hif-1 and contributes to growth and angiogenesis of glioblastoma. Cell Biochem. Biophys..

[25] Xu C., Liu Y., Xiao L., Guo C., Deng S., Zheng S., Zeng E. (2016). The involvement of anterior gradient 2 in the stromal cell-derived factor 1-induced epithelial-mesenchymal transition of glioblastoma. Tumor Biol..

[26] Obacz J., Brychtova V., Podhorec J., Fabian P., Dobes P., Vojtesek B., Hrstka R. (2015). Anterior gradient protein 3 is associated with less aggressive tumors and better outcome of breast cancer patients. Oncotargets Ther..

[27] Gray T.A., MacLaine N.J., Michie C.O., Bouchalova P., Murray E., Howie J., Hrstka R., Maslon M.M., Nenutil R., Vojtesek B. (2012). Anterior gradient-3: A novel biomarker for ovarian cancer that mediates cisplatin resistance in xenograft models. J. Immunol. Methods.

[28] Obacz J., Takacova M., Brychtova V., Dobes P., Pastorekova S., Vojtesek B., Hrstka R. (2015). The role of agr2 and agr3 in cancer: Similar but not identical. Eur. J. Cell Biol..

[29] Petek E., Windpassinger C., Egger H., Kroisel P.M., Wagner K. (2000). Localization of the human anterior gradient-2 gene (agr2) to chromosome band 7p21.3 by radiation hybrid mapping and fluorescencein situ hybridisation. Cytogenet. Cell Genet..

[30] Kovalev L.I., Shishkin S.S., Khasigov P.Z., Dzeranov N.K., Kazachenko A.V., Toropygin I., Mamykina S.V. (2006). Identification of agr2 protein, a novel potential cancer marker, using proteomics technologies. Prikladnaia Biokhimiia I Mikrobiologiia.

[31] Ahmad R., Nicora C.D., Shukla A.K., Smith R.D., Qian W.J., Liu A.Y. (2016). An efficient method for native protein purification in the selected range from prostate cancer tissue digests. Chin. Clin. Oncol..

[32] Neeb A., Hefele S., Bormann S., Parson W., Adams F., Wolf P., Miernik A., Schoenthaler M., Kroenig M., Wilhelm K. (2014). Splice variant transcripts of the anterior gradient 2 gene as a marker of prostate cancer. Oncotarget.

[33] Gupta A., Wodziak D., Tun M., Bouley D.M., Lowe A.W. (2013). Loss of anterior gradient 2 (agr2) expression results in hyperplasia and defective lineage maturation in the murine stomach. J. Biol. Chem..

[34] Shishkin S.S., Eremina L.S., Kovalev L.I., Kovaleva M.A. (2013). Agr2, erp57/grp58, and some other human protein disulfide isomerases. Biochemistry.

[35] Gray T.A., Murray E., Nowicki M.W., Remnant L., Scherl A., Muller P., Vojtesek B., Hupp T.R. (2013). Development of a fluorescent monoclonal antibody-based assay to measure the allosteric effects of synthetic peptides on self-oligomerization of agr2 protein. Protein Sci..

[36] Clarke C., Rudland P., Barraclough R. (2015). The metastasis-inducing protein agr2 is o-glycosylated upon secretion from mammary epithelial cells. Mol. Cell. Biochem..

[37] Fagerberg L., Hallstrom B.M., Oksvold P., Kampf C., Djureinovic D., Odeberg J., Habuka M., Tahmasebpoor S., Danielsson A., Edlund K. (2014). Analysis of the human tissue-specific expression by genome-wide integration of transcriptomics and antibody-based proteomics. Mol. Cell. Proteom..

[38] Riener M.O., Thiesler T., Hellerbrand C., Amann T., Cathomas G., Fritzsche F.R., Dahl E., Bahra M., Weichert W., Terracciano L. (2014). Loss of anterior gradient-2 expression is an independent prognostic factor in colorectal carcinomas. Eur. J. Cancer.

[39] Di Maro G., Salerno P., Unger K., Orlandella F.M., Monaco M., Chiappetta G., Thomas G., Oczko-Wojciechowska M., Masullo M., Jarzab B. (2014). Anterior gradient protein 2 promotes survival, migration and invasion of papillary thyroid carcinoma cells. Mol. Cancer.

[40] Uhlen M., Fagerberg L., Hallstrom B.M., Lindskog C., Oksvold P., Mardinoglu A., Sivertsson A., Kampf C., Sjostedt E., Asplund A. (2015). Proteomics. Tissue-based map of the human proteome. Science.

[41] Thul P.J., Akesson L., Wiking M., Mahdessian D., Geladaki A., Ait Blal H., Alm T., Asplund A., Bjork L., Breckels L.M. (2017). A subcellular map of the human proteome. Science.

[42] Uhlen M., Zhang C., Lee S., Sjostedt E., Fagerberg L., Bidkhori G., Benfeitas R., Arif M., Liu Z., Edfors F. (2017). A pathology atlas of the human cancer transcriptome. Science.

[43] Innes H.E., Liu D., Barraclough R., Davies M.P., O’Neill P.A., Platt-Higgins A., de Silva Rudland S., Sibson D.R., Rudland P.S. (2006). Significance of the metastasis-inducing protein agr2 for outcome in hormonally treated breast cancer patients. Br. J. Cancer.

[44] Ondrouskova E., Sommerova L., Nenutil R., Coufal O., Bouchal P., Vojtesek B., Hrstka R. (2017). Agr2 associates with her2 expression predicting poor outcome in subset of estrogen receptor negative breast cancer patients. Exp. Mol. Pathol..

[45] Guo J., Gong G., Zhang B. (2017). Identification and prognostic value of anterior gradient protein 2 expression in breast cancer based on tissue microarray. Tumour Biol..

[46] Ramachandran V., Arumugam T., Wang H., Logsdon C.D. (2008). Anterior gradient 2 is expressed and secreted during the development of pancreatic cancer and promotes cancer cell survival. Cancer Res..

[47] Riener M.O., Pilarsky C., Gerhardt J., Grutzmann R., Fritzsche F.R., Bahra M., Weichert W., Kristiansen G. (2009). Prognostic significance of agr2 in pancreatic ductal adenocarcinoma. Histol. Histopathol..

[48] Mizuuchi Y., Aishima S., Ohuchida K., Shindo K., Fujino M., Hattori M., Miyazaki T., Mizumoto K., Tanaka M., Oda Y. (2015). Anterior gradient 2 downregulation in a subset of pancreatic ductal adenocarcinoma is a prognostic factor indicative of epithelial-mesenchymal transition. Lab. Investig..

[49] Uthaisar K., Vaeteewoottacharn K., Seubwai W., Talabnin C., Sawanyawisuth K., Obchoei S., Kraiklang R., Okada S., Wongkham S. (2016). Establishment and characterization of a novel human cholangiocarcinoma cell line with high metastatic activity. Oncol. Rep..

[50] Vivekanandan P., Micchelli S.T., Torbenson M. (2009). Anterior gradient-2 is overexpressed by fibrolamellar carcinomas. Hum. Pathol..

[51] Lepreux S., Bioulac-Sage P., Chevet E. (2011). Differential expression of the anterior gradient protein-2 is a conserved feature during morphogenesis and carcinogenesis of the biliary tree. Liver Int..

[52] Maresh E.L., Mah V., Alavi M., Horvath S., Bagryanova L., Liebeskind E.S., Knutzen L.A., Zhou Y., Chia D., Liu A.Y. (2010). Differential expression of anterior gradient gene agr2 in prostate cancer. BMC Cancer.

[53] Wayner E.A., Quek S.I., Ahmad R., Ho M.E., Loprieno M.A., Zhou Y., Ellis W.J., True L.D., Liu A.Y. (2012). Development of an elisa to detect the secreted prostate cancer biomarker agr2 in voided urine. Prostate.

[54] Shi T., Gao Y., Quek S.I., Fillmore T.L., Nicora C.D., Su D., Zhao R., Kagan J., Srivastava S., Rodland K.D. (2014). A highly sensitive targeted mass spectrometric assay for quantification of agr2 protein in human urine and serum. J. Proteome Res..

[55] Rice G.E., Edgell T.A., Autelitano D.J. (2010). Evaluation of midkine and anterior gradient 2 in a multimarker panel for the detection of ovarian cancer. J. Exp. Clin. Cancer Res..

[56] Kamal A., Valentijn A., Barraclough R., Rudland P., Rahmatalla N., Martin-Hirsch P., Stringfellow H., Decruze S.B., Hapangama D.K. (2018). High agr2 protein is a feature of low grade endometrial cancer cells. Oncotarget.

[57] Fritzsche F.R., Dahl E., Dankof A., Burkhardt M., Pahl S., Petersen I., Dietel M., Kristiansen G. (2007). Expression of agr2 in non small cell lung cancer. Histol. Histopathol..

[58] Pizzi M., Fassan M., Balistreri M., Galligioni A., Rea F., Rugge M. (2012). Anterior gradient 2 overexpression in lung adenocarcinoma. Appl. Immunohistochem. Mol. Morphol..

[59] Chung K., Nishiyama N., Wanibuchi H., Yamano S., Hanada S., Wei M., Suehiro S., Kakehashi A. (2012). Agr2 as a potential biomarker of human lung adenocarcinoma. Osaka City Med. J..

[60] Narumi S., Miki Y., Hata S., Ebina M., Saito M., Mori K., Kobayashi M., Suzuki T., Iwabuchi E., Sato I. (2015). Anterior gradient 2 is correlated with egfr mutation in lung adenocarcinoma tissues. Int. J. Biol. Markers.

[61] Tomoshige K., Guo M., Tsuchiya T., Fukazawa T., Fink-Baldauf I.M., Stuart W.D., Naomoto Y., Nagayasu T., Maeda Y. (2018). An egfr ligand promotes egfr-mutant but not kras-mutant lung cancer in vivo. Oncogene.

[62] Alavi M., Mah V., Maresh E.L., Bagryanova L., Horvath S., Chia D., Goodglick L., Liu A.Y. (2015). High expression of agr2 in lung cancer is predictive of poor survival. BMC Cancer.

[63] Li Y., Wang W., Liu Z., Jiang Y., Lu J., Xie H., Tang F. (2018). Agr2 diagnostic value in nasopharyngeal carcinoma prognosis. Clin. Chim. Acta.

[64] Pizzi M., Fassan M., Realdon S., Balistreri M., Battaglia G., Giacometti C., Zaninotto G., Zagonel V., De Boni M., Rugge M. (2012). Anterior gradient 2 profiling in barrett columnar epithelia and adenocarcinoma. Hum. Pathol..

[65] Zhang J., Jin Y., Xu S., Zheng J., Zhang Q.I., Wang Y., Chen J., Huang Y., He X., Zhao Z. (2016). Agr2 is associated with gastric cancer progression and poor survival. Oncol. Lett..

[66] Valladares-Ayerbes M., Blanco-Calvo M., Reboredo M., Lorenzo-Patino M.J., Iglesias-Diaz P., Haz M., Diaz-Prado S., Medina V., Santamarina I., Pertega S. (2012). Evaluation of the adenocarcinoma-associated gene agr2 and the intestinal stem cell marker lgr5 as biomarkers in colorectal cancer. Int. J. Mol. Sci..

[67] Kim S.J., Jun S., Cho H.Y., Lee D.C., Yeom Y.I., Kim J.H., Kang D. (2014). Knockdown of anterior gradient 2 expression extenuates tumor-associated phenotypes of snu-478 ampulla of vater cancer cells. BMC Cancer.

[68] Kim S.J., Kim D.H., Kang D., Kim J.H. (2014). Expression of anterior gradient 2 is decreased with the progression of human biliary tract cancer. Tohoku J. Exp. Med..

[69] Ho M.E., Quek S.I., True L.D., Seiler R., Fleischmann A., Bagryanova L., Kim S.R., Chia D., Goodglick L., Shimizu Y. (2016). Bladder cancer cells secrete while normal bladder cells express but do not secrete agr2. Oncotarget.

[70] Ma S.R., Wang W.M., Huang C.F., Zhang W.F., Sun Z.J. (2015). Anterior gradient protein 2 expression in high grade head and neck squamous cell carcinoma correlated with cancer stem cell and epithelial mesenchymal transition. Oncotarget.

[71] Tohti M., Li J., Ma C., Li W., Lu Z., Hu Y. (2015). Expression of agr2 in pituitary adenomas and its association with tumor aggressiveness. Oncol. Lett..

[72] Tohti M., Li J., Tang C., Wen G., Abdujilil A., Yizim P., Ma C. (2017). Serum agr2 as a useful biomarker for pituitary adenomas. Clin. Neurol. Neurosurg..

[73] Vitello E.A., Quek S.I., Kincaid H., Fuchs T., Crichton D.J., Troisch P., Liu A.Y. (2016). Cancer-secreted agr2 induces programmed cell death in normal cells. Oncotarget.

[74] Hrstka R., Murray E., Brychtova V., Fabian P., Hupp T.R., Vojtesek B. (2013). Identification of an akt-dependent signalling pathway that mediates tamoxifen-dependent induction of the pro-metastatic protein anterior gradient-2. Cancer Lett..

[75] Zhang Y., Ali T.Z., Zhou H., D’Souza D.R., Lu Y., Jaffe J., Liu Z., Passaniti A., Hamburger A.W. (2010). Erbb3 binding protein 1 represses metastasis-promoting gene anterior gradient protein 2 in prostate cancer. Cancer Res..

[76] Zheng W., Rosenstiel P., Huse K., Sina C., Valentonyte R., Mah N., Zeitlmann L., Grosse J., Ruf N., Nurnberg P. (2006). Evaluation of agr2 and agr3 as candidate genes for inflammatory bowel disease. Genes Immun..

[77] Milewski D., Balli D., Ustiyan V., Le T., Dienemann H., Warth A., Breuhahn K., Whitsett J.A., Kalinichenko V.V., Kalin T.V. (2017). Foxm1 activates agr2 and causes progression of lung adenomas into invasive mucinous adenocarcinomas. PLoS Genet..

[78] Broustas C.G., Hopkins K.M., Panigrahi S.K., Wang L., Virk R.K., Lieberman H.B. (2019). Rad9a promotes metastatic phenotypes through transcriptional regulation of anterior gradient 2 (agr2). Carcinogenesis.

[79] Sommerova L., Ondrouskova E., Vojtesek B., Hrstka R. (2017). Suppression of agr2 in a tgf-beta-induced smad regulatory pathway mediates epithelial-mesenchymal transition. BMC Cancer.

[80] Norris A.M., Gore A., Balboni A., Young A., Longnecker D.S., Korc M. (2013). Agr2 is a smad4-suppressible gene that modulates muc1 levels and promotes the initiation and progression of pancreatic intraepithelial neoplasia. Oncogene.

[81] Tu M.J., Pan Y.Z., Qiu J.X., Kim E.J., Yu A.M. (2016). Microrna-1291 targets the foxa2-agr2 pathway to suppress pancreatic cancer cell proliferation and tumorigenesis. Oncotarget.

[82] Pan B., Yang J., Wang X., Xu K., Ikezoe T. (2018). Mir-217 sensitizes chronic myelogenous leukemia cells to tyrosine kinase inhibitors by targeting pro-oncogenic anterior gradient 2. Exp. Hematol..

[83] Xue X., Fei X., Hou W., Zhang Y., Liu L., Hu R. (2018). Mir-342-3p suppresses cell proliferation and migration by targeting agr2 in non-small cell lung cancer. Cancer Lett..

[84] Maslon M.M., Hrstka R., Vojtesek B., Hupp T.R. (2010). A divergent substrate-binding loop within the pro-oncogenic protein anterior gradient-2 forms a docking site for reptin. J. Mol. Biol..

[85] Park K., Chung Y.J., So H., Kim K., Park J., Oh M., Jo M., Choi K., Lee E.J., Choi Y.L. (2011). Agr2, a mucinous ovarian cancer marker, promotes cell proliferation and migration. Exp. Mol. Med..

[86] Dumartin L., Alrawashdeh W., Trabulo S.M., Radon T.P., Steiger K., Feakins R.M., di Magliano M.P., Heeschen C., Esposito I., Lemoine N.R. (2017). Er stress protein agr2 precedes and is involved in the regulation of pancreatic cancer initiation. Oncogene.

[87] Gupta A., Dong A., Lowe A.W. (2012). Agr2 gene function requires a unique endoplasmic reticulum localization motif. J. Biol. Chem..

[88] Vanderlaag K.E., Hudak S., Bald L., Fayadat-Dilman L., Sathe M., Grein J., Janatpour M.J. (2010). Anterior gradient-2 plays a critical role in breast cancer cell growth and survival by modulating cyclin d1, estrogen receptor-alpha and survivin. Breast Cancer Res..

[89] Guo H., Zhu Q., Yu X., Merugu S.B., Mangukiya H.B., Smith N., Li Z., Zhang B., Negi H., Rong R. (2017). Tumor-secreted anterior gradient-2 binds to vegf and fgf2 and enhances their activities by promoting their homodimerization. Oncogene.

[90] Jia M., Guo Y., Zhu D., Zhang N., Li L., Jiang J., Dong Y., Xu Q., Zhang X., Wang M. (2018). Pro-metastatic activity of agr2 interrupts angiogenesis target bevacizumab efficiency via direct interaction with vegfa and activation of nf-kappab pathway. Biochim. Biophys. Acta Mol. Basis Dis..

[91] Sung H.Y., Choi E.N., Lyu D., Park A.K., Ju W., Ahn J.H. (2014). Aberrant hypomethylation-mediated agr2 overexpression induces an aggressive phenotype in ovarian cancer cells. Oncol. Rep..

[92] Dumartin L., Whiteman H.J., Weeks M.E., Hariharan D., Dmitrovic B., Iacobuzio-Donahue C.A., Brentnall T.A., Bronner M.P., Feakins R.M., Timms J.F. (2011). Agr2 is a novel surface antigen that promotes the dissemination of pancreatic cancer cells through regulation of cathepsins b and d. Cancer Res..

[93] Chanda D., Lee J.H., Sawant A., Hensel J.A., Isayeva T., Reilly S.D., Siegal G.P., Smith C., Grizzle W., Singh R. (2014). Anterior gradient protein-2 is a regulator of cellular adhesion in prostate cancer. PLoS ONE.

[94] Park S.W., Zhen G., Verhaeghe C., Nakagami Y., Nguyenvu L.T., Barczak A.J., Killeen N., Erle D.J. (2009). The protein disulfide isomerase agr2 is essential for production of intestinal mucus. Proc. Natl. Acad. Sci. USA.

[95] Bhatia R., Gautam S.K., Cannon A., Thompson C., Hall B.R., Aithal A., Banerjee K., Jain M., Solheim J.C., Kumar S. (2019). Cancer-associated mucins: Role in immune modulation and metastasis. Cancer Metastasis Rev..

[96] Reynolds I.S., Fichtner M., McNamara D.A., Kay E.W., Prehn J.H.M., Burke J.P. (2019). Mucin glycoproteins block apoptosis; promote invasion, proliferation, and migration; and cause chemoresistance through diverse pathways in epithelial cancers. Cancer Metastasis Rev..

[97] Tsuji T., Satoyoshi R., Aiba N., Kubo T., Yanagihara K., Maeda D., Goto A., Ishikawa K., Yashiro M., Tanaka M. (2015). Agr2 mediates paracrine effects on stromal fibroblasts that promote invasion by gastric signet-ring carcinoma cells. Cancer Res..

[98] Yosudjai J., Inpad C., Chomwong S., Dana P., Sawanyawisuth K., Phimsen S., Wongkham S., Jirawatnotai S., Kaewkong W. (2018). An aberrantly spliced isoform of anterior gradient-2, agr2vh promotes migration and invasion of cholangiocarcinoma cell. Biomed. Pharmacother..

[99] Fessart D., Domblides C., Avril T., Eriksson L.A., Begueret H., Pineau R., Malrieux C., Dugot-Senant N., Lucchesi C., Chevet E. (2016). Secretion of protein disulphide isomerase agr2 confers tumorigenic properties. eLife.

[100] Kim J., Chung J.Y., Kim T.J., Lee J.W., Kim B.G., Bae D.S., Choi C.H., Hewitt S.M. (2018). Genomic network-based analysis reveals pancreatic adenocarcinoma up-regulating factor-related prognostic markers in cervical carcinoma. Front. Oncol..

[101] Delom F., Nazaraliyev A., Fessart D. (2018). The role of protein disulphide isomerase agr2 in the tumour niche. Biol. Cell.

[102] Hrstka R., Brychtova V., Fabian P., Vojtesek B., Svoboda M. (2013). Agr2 predicts tamoxifen resistance in postmenopausal breast cancer patients. Dis. Markers.

[103] Hrstka R., Podhorec J., Nenutil R., Sommerova L., Obacz J., Durech M., Faktor J., Bouchal P., Skoupilova H., Vojtesek B. (2017). Tamoxifen-dependent induction of agr2 is associated with increased aggressiveness of endometrial cancer cells. Cancer Investig..

[104] Li Z., Zhu Q., Hu L., Chen H., Wu Z., Li D. (2015). Anterior gradient 2 is a binding stabilizer of hypoxia inducible factor-1alpha that enhances cocl2 -induced doxorubicin resistance in breast cancer cells. Cancer Sci..

[105] Wu J., Wang C., Li X., Song Y., Wang W., Li C., Hu J., Zhu Z., Li J., Zhang W. (2012). Identification, characterization and application of a g-quadruplex structured DNA aptamer against cancer biomarker protein anterior gradient homolog 2. PLoS ONE.

[106] Wu Z.H., Zhu Q., Gao G.W., Zhou C.C., Li D.W. (2010). Preparation, characterization and potential application of monoclonal antibody 18a4 against agr2. Chin. J. Cell. Mol. Immunol..

[107] Lee H.J., Hong C.Y., Kim M.H., Lee Y.K., Nguyen-Pham T.N., Park B.C., Yang D.H., Chung I.J., Kim H.J., Lee J.J. (2012). In vitro induction of anterior gradient-2-specific cytotoxic t lymphocytes by dendritic cells transduced with recombinant adenoviruses as a potential therapy for colorectal cancer. Exp. Mol. Med..

[108] Lee H.J., Hong C.Y., Jin C.J., Kim M.H., Lee Y.K., Nguyen-Pham T.N., Lee H., Park B.C., Chung I.J., Kim H.J. (2012). Identification of novel hla-a*0201-restricted epitopes from anterior gradient-2 as a tumor-associated antigen against colorectal cancer. Cell. Mol. Immunol..

[109] Garri C., Howell S., Tiemann K., Tiffany A., Jalali-Yazdi F., Alba M.M., Katz J.E., Takahashi T.T., Landgraf R., Gross M.E. (2018). Identification, characterization and application of a new peptide against anterior gradient homolog 2 (agr2). Oncotarget.

[110] Arumugam T., Deng D., Bover L., Wang H., Logsdon C.D., Ramachandran V. (2015). New blocking antibodies against novel agr2-c4.4a pathway reduce growth and metastasis of pancreatic tumors and increase survival in mice. Mol. Cancer Ther..

[111] Guo H., Chen H., Zhu Q., Yu X., Rong R., Merugu S.B., Mangukiya H.B., Li D. (2016). A humanized monoclonal antibody targeting secreted anterior gradient 2 effectively inhibits the xenograft tumor growth. Biochem. Biophys. Res. Commun..

[112] Pan F., Li W., Yang W., Yang X.Y., Liu S., Li X., Zhao X., Ding H., Qin L., Pan Y. (2018). Anterior gradient 2 as a supervisory marker for tumor vessel normalization induced by anti-angiogenic treatment. Oncol. Lett..

[113] Barrett T., Wilhite S.E., Ledoux P., Evangelista C., Kim I.F., Tomashevsky M., Marshall K.A., Phillippy K.H., Sherman P.M., Holko M. (2013). Ncbi geo: Archive for functional genomics data sets–update. Nucleic Acids Res..

[114] Kolesnikov N., Hastings E., Keays M., Melnichuk O., Tang Y.A., Williams E., Dylag M., Kurbatova N., Brandizi M., Burdett T. (2015). Arrayexpress update—Simplifying data submissions. Nucleic Acids Res..

[115] Ritchie M.E., Phipson B., Wu D., Hu Y., Law C.W., Shi W., Smyth G.K. (2015). *Limma* powers differential expression analyses for RNA-sequencing and microarray studies. Nucleic Acids Res..

[116] Holsken A., Sill M., Merkle J., Schweizer L., Buchfelder M., Flitsch J., Fahlbusch R., Metzler M., Kool M., Pfister S.M. (2016). Adamantinomatous and papillary craniopharyngiomas are characterized by distinct epigenomic as well as mutational and transcriptomic profiles. Acta Neuropathol. Commun..

[117] Lee Y., Liu J., Patel S., Cloughesy T., Lai A., Farooqi H., Seligson D., Dong J., Liau L., Becker D. (2010). Genomic landscape of meningiomas. Brain Pathol..

[118] Gump J.M., Donson A.M., Birks D.K., Amani V.M., Rao K.K., Griesinger A.M., Kleinschmidt-DeMasters B.K., Johnston J.M., Anderson R.C., Rosenfeld A. (2015). Identification of targets for rational pharmacological therapy in childhood craniopharyngioma. Acta Neuropathol. Commun..

[119] Michaelis K.A., Knox A.J., Xu M., Kiseljak-Vassiliades K., Edwards M.G., Geraci M., Kleinschmidt-DeMasters B.K., Lillehei K.O., Wierman M.E. (2011). Identification of growth arrest and DNA-damage-inducible gene beta (gadd45beta) as a novel tumor suppressor in pituitary gonadotrope tumors. Endocrinology.

[120] Zhou G., Soufan O., Ewald J., Hancock R.E.W., Basu N., Xia J. (2019). Networkanalyst 3.0: A visual analytics platform for comprehensive gene expression profiling and meta-analysis. Nucleic Acids Res..

[121] Xia J., Gill E.E., Hancock R.E. (2015). Networkanalyst for statistical, visual and network-based meta-analysis of gene expression data. Nat. Protoc..

[122] Vazquez-Sanchez A.Y., Hinojosa L.M., Parraguirre-Martinez S., Gonzalez A., Morales F., Montalvo G., Vera E., Hernandez-Gallegos E., Camacho J. (2018). Expression of katp channels in human cervical cancer: Potential tools for diagnosis and therapy. Oncol. Lett..

[123] Chang Y.C., Chan Y.C., Chang W.M., Lin Y.F., Yang C.J., Su C.Y., Huang M.S., Wu A.T.H., Hsiao M. (2017). Feedback regulation of aldoa activates the hif-1alpha/mmp9 axis to promote lung cancer progression. Cancer Lett..

[124] Musgrove E.A., Caldon C.E., Barraclough J., Stone A., Sutherland R.L. (2011). Cyclin d as a therapeutic target in cancer. Nat. Rev. Cancer.

[125] Inoue K., Fry E.A. (2018). Aberrant expression of p16(ink4a) in human cancers—A new biomarker?. Cancer Rep. Rev..

[126] Olar A., Goodman L.D., Wani K.M., Boehling N.S., Sharma D.S., Mody R.R., Gumin J., Claus E.B., Lang F.F., Cloughesy T.F. (2018). A gene expression signature predicts recurrence-free survival in meningioma. Oncotarget.

[127] Qian C., Xia Y., Ren Y., Yin Y., Deng A. (2017). Identification and validation of psat1 as a potential prognostic factor for predicting clinical outcomes in patients with colorectal carcinoma. Oncol. Lett..

[128] Jiang J., Zhang L., Chen H., Lei Y., Zhang T., Wang Y., Jin P., Lan J., Zhou L., Huang Z. (2019). Regorafenib induces lethal autophagy arrest by stabilizing psat1 in glioblastoma. Autophagy.

[129] Tabernero M.D., Maillo A., Gil-Bellosta C.J., Castrillo A., Sousa P., Merino M., Orfao A. (2009). Gene expression profiles of meningiomas are associated with tumor cytogenetics and patient outcome. Brain Pathol..

[130] Jandial R., Choy C., Levy D.M., Chen M.Y., Ansari K.I. (2017). Astrocyte-induced reelin expression drives proliferation of her2(+) breast cancer metastases. Clin. Exp. Metastasis.

[131] Lin L., Yan F., Zhao D., Lv M., Liang X., Dai H., Qin X., Zhang Y., Hao J., Sun X. (2016). Reelin promotes the adhesion and drug resistance of multiple myeloma cells via integrin beta1 signaling and stat3. Oncotarget.

[132] Wu X., Giobbie-Hurder A., Liao X., Connelly C., Connolly E.M., Li J., Manos M.P., Lawrence D., McDermott D., Severgnini M. (2017). Angiopoietin-2 as a biomarker and target for immune checkpoint therapy. Cancer Immunol. Res..

[133] Li Y.H., Yu C.Y., Li X.X., Zhang P., Tang J., Yang Q., Fu T., Zhang X., Cui X., Tu G. (2018). Therapeutic target database update 2018: Enriched resource for facilitating bench-to-clinic research of targeted therapeutics. Nucleic Acids Res..

[134] Schulten H.J., Hussein D., Al-Adwani F., Karim S., Al-Maghrabi J., Al-Sharif M., Jamal A., Al-Ghamdi F., Baeesa S.S., Bangash M. (2016). Microarray expression data identify dcc as a candidate gene for early meningioma progression. PLoS ONE.

[135] Vassilopoulos A., Xiao C., Chisholm C., Chen W., Xu X., Lahusen T.J., Bewley C., Deng C.X. (2014). Synergistic therapeutic effect of cisplatin and phosphatidylinositol 3-kinase (pi3k) inhibitors in cancer growth and metastasis of brca1 mutant tumors. J. Biol. Chem..

[136] Schulten H.J., Hussein D. (2019). Array expression meta-analysis of cancer stem cell genes identifies upregulation of podxl especially in dcc low expression meningiomas. PLoS ONE.

[137] Xu C., Sun L., Jiang C., Zhou H., Gu L., Liu Y., Xu Q. (2017). Spp1, analyzed by bioinformatics methods, promotes the metastasis in colorectal cancer by activating emt pathway. Biomed. Pharmacother..

[138] Seo K.J., Kim M., Kim J. (2015). Prognostic implications of adhesion molecule expression in colorectal cancer. Int. J. Clin. Exp. Pathol..

[139] Hulleman E., Quarto M., Vernell R., Masserdotti G., Colli E., Kros J.M., Levi D., Gaetani P., Tunici P., Finocchiaro G. (2009). A role for the transcription factor hey1 in glioblastoma. J. Cell. Mol. Med..

[140] Salvi S., Bonafe M., Bravaccini S. (2019). Androgen receptor in breast cancer: A wolf in sheep’s clothing? A lesson from prostate cancer. Semin. Cancer Biol..

[141] Thike A.A., Yong-Zheng Chong L., Cheok P.Y., Li H.H., Wai-Cheong Yip G., Huat Bay B., Tse G.M., Iqbal J., Tan P.H. (2014). Loss of androgen receptor expression predicts early recurrence in triple-negative and basal-like breast cancer. Mod. Pathol..

[142] Schroeder A., Herrmann A., Cherryholmes G., Kowolik C., Buettner R., Pal S., Yu H., Muller-Newen G., Jove R. (2014). Loss of androgen receptor expression promotes a stem-like cell phenotype in prostate cancer through stat3 signaling. Cancer Res..

[143] Valkenburg K.C., De Marzo A.M., Williams B.O. (2017). Deletion of tumor suppressors adenomatous polyposis coli and smad4 in murine luminal epithelial cells causes invasive prostate cancer and loss of androgen receptor expression. Oncotarget.

[144] Breier G., Grosser M., Rezaei M. (2014). Endothelial cadherins in cancer. Cell Tissue Res..

[145] Zanetta L., Corada M., Grazia Lampugnani M., Zanetti A., Breviario F., Moons L., Carmeliet P., Pepper M.S., Dejana E. (2005). Downregulation of vascular endothelial-cadherin expression is associated with an increase in vascular tumor growth and hemorrhagic complications. Thromb. Haemost..

[146] Rezaei M., Friedrich K., Wielockx B., Kuzmanov A., Kettelhake A., Labelle M., Schnittler H., Baretton G., Breier G. (2012). Interplay between neural-cadherin and vascular endothelial-cadherin in breast cancer progression. Breast Cancer Res..

[147] Evans D.R., Venkitachalam S., Revoredo L., Dohey A.T., Clarke E., Pennell J.J., Powell A.E., Quinn E., Ravi L., Gerken T.A. (2018). Evidence for galnt12 as a moderate penetrance gene for colorectal cancer. Hum. Mutat..

